# Clinical efficacy of bioactive restorative materials in controlling secondary caries: a systematic review and network meta-analysis

**DOI:** 10.1186/s12903-023-03110-y

**Published:** 2023-06-15

**Authors:** Noeleni Souza Pinto, Gabriela Rebouças Jorge, Jader Vasconcelos, Livia Fernandes Probst, Alessandro Diogo De-Carli, Andrea Freire

**Affiliations:** 1 School of Dentistry, Universidade Federal de Mato Grosso Do Sul, Av. Costa E Silva, S/N, Universitário, Campo Grande, MS 79070-900 Brazil; 2 Secretaria de Saúde (SESAU), Campo Grande, Brazil; 3 Unidade de Avaliação de Tecnologias Em Saúde, Hospital Alemão Oswaldo Cruz, São Paulo, Brazil

**Keywords:** Dental caries, Secondary caries, Resin composite, Amalgam, Bioactive materials

## Abstract

**Background:**

This systematic review and network meta-analysis aimed to compare the clinical efficacy of bioactive and conventional restorative materials in controlling secondary caries (SC) and to provide a classification of these materials according to their effectiveness.

**Methods:**

A search was performed in Pubmed, Web of Science, Embase, BBO, Lilacs, Cochrane Library, Scopus, IBECS and gray literature. Clinical trials were included, with no language or publication date limitations. Paired and network meta-analyses were performed with random-effects models, comparing treatments of interest and classifying them according to effectiveness in the permanent and deciduous dentition and at 1-year or 2/more years of follow-up. The risk of bias and certainty of evidence were evaluated.

**Results:**

Sixty-two studies were included in the qualitative syntheses and 39 in the quantitative ones. In permanent teeth, resin composite (RC) (RR = 2.00; 95%CI = 1.10, 3.64) and amalgam (AAG) (RR = 1.79; 95%CI = 1.04, 3.09) showed a higher risk of SC than Glass Ionomer Cement (GIC). In the deciduous teeth, however, a higher risk of SC was observed with RC than with AAG (RR = 2.46; 95%CI = 1.42, 4.27) and in GIC when compared to Resin-Modified Glass Ionomer Cement (RMGIC = 1.79; 95%CI = 1.04, 3.09). Most randomized clinical trials studies showed low or moderate risk of bias.

**Conclusion:**

There is a difference between bioactive restorative materials for SC control, with GIC being more effective in the permanent teeth and the RMGIC in the deciduous teeth. Bioactive restorative materials can be adjuvants in the control of SC in patients at high risk for caries.

**Supplementary Information:**

The online version contains supplementary material available at 10.1186/s12903-023-03110-y.

## Background

Dental caries or tooth decay is considered a complex and polymicrobial dysbiosis, resulting from an imbalance in the demineralization (DEM) and remineralization (REM) process [1]. Commensal microorganisms are able to metabolize carbohydrates and produce acids that can initiate tooth structure DEM. In individuals on a low-sugar diet, a physiological mechanism such as salivation is capable of rebalancing the pH and interrupting the caries progression [2], favoring REM. However, when the individual has a high intake of sugar, there is a microbial imbalance in the oral environment, favoring the biofilm acidification as a result of carbohydrate metabolism, and consequently, DEM [3], if this biofilm is not disorganized and remains stagnant in the dental tissues.

The same process can occur between restoration and cavity preparation, resulting in secondary caries (SC) [4]. The margins of restorations can be considered critical areas due to the possible presence of marginal gaps produced by polymerization contraction, porosity or fractures [5]. In these situations, the accumulation of biofilm is facilitated, making restorations prone to faster degradation, which may lead to the formation of caries lesions [6]. SC rates for polymeric restorative materials are very high, around approximately 60%, and have been identified as one of the main reasons for failure and replacement of resin composite restorations [7–9].

To decrease the replacement rate of restorations due to SC, smart and alternative restorative materials have been developed [10, 11], which can act in the DEM-REM process. The complexity of oral biofilms contributes to the difficulty in developing new effective dental materials. Therefore, new technologies have been explored to develop bioactive dental materials to reduce or modulate bacterial activities related to caries [4]. Bioactive materials can be either natural or synthetic, and are intended to repair, regenerate or replace tissues [12]. The ions most frequently associated with increased resistance of dental tissue to acid attack are calcium, phosphate and fluorides, which may come from the saliva or external sources [11, 13]. Calcium phosphate in the saliva is the natural defense against tooth mineral loss. Fluoride is considered an additional method of controlling the DEM-REM process [13].

New composites have been manufactured with the purpose of inhibiting SC, neutralizing acids and replacing lost minerals [14, 15]. The new class of hybrids, Glass ionomer cements (GICs), or Giomers, are one of the latest in the field of fluoride-releasing restorative materials. These were obtained by combining resin composite and glass ionomer cements (GICs), resulting in composites that offer aesthetic and functional results, as well as protection against caries, due to the incorporation of pre-reacted glass particles [16].

Moreover, the use of fluoride is one of the most effective methods for controlling the DEM-REM process [5, 17]. Its effect reduces DEM, increases REM, interferes with biofilm formation, inhibits microbial growth and metabolic processes [5, 17]. This physical–chemical mechanism occurs every time sugar is ingested, and the biofilm pH decreases. When sugar intake ceases and the pH rises again, the fluoride present in oral fluids increases REM [17, 18].

Aiming to provide fluoride at SC risk sites, fluoride-containing restorative materials have been developed, including glass-ionomer cements, resin-modified glass-ionomer cements, compomers, composites, and amalgams. The antibacterial and cariostatic properties of restorative materials are often associated with the amount of fluoride released, which is substantial only immediately after the material is used. However, materials that release fluoride can act as a fluoride reservoir, which is replenished after the topical use [19, 20]. Despite the cariostatic effect obtained from the increase in fluoride content, clinical studies have shown conflicting data on the magnitude at which these materials act in the process of controlling SC progression, when compared to non-fluoride restorative materials [20].

The aim of this study was to compare the effectiveness of bioactive restorative materials based on calcium, phosphate and fluoride and conventional restorative materials in controlling SC and to provide a classification of these materials according to their effectiveness.

## Methods

### Protocol and registration

This systematic review followed the recommendations of the Cochrane Handbook for Systematic Reviews of Interventions. It was reported in accordance with the PRISMA Extension Statement for Reporting of Systematic Reviews Incorporating Network Meta-analyses of Health Care Interventions (PRISMA-NMA) [21]. The protocol was registered in the International Prospective Register of Systematic Reviews (PROSPERO) database, under protocol number CRD42020137298.

### Eligibility criteria

The inclusion criteria were defined based on the PICOS strategy: Population: participants with indication for dental restoration; Intervention: bioactive restorative materials including calcium, phosphate or fluoride (Ca, PO_4_, F) in their composition; Comparison: conventional restorative materials that do not have any of the three above mentioned compounds in their formulation; Outcome: secondary caries; Study design: clinical trial.

Restorative materials whose composition differed from calcium, phosphate or fluoride were excluded; as well as clinical case reports, literature reviews, case series, pilot studies, editorial letters, observational and descriptive studies.

### Information sources

The electronic search strategies were performed in the following databases: Pubmed, Web of Science, Embase, BBO, Lilacs, Cochrane Library, Scopus and IBECS databases. Studies were identified by other methods—Google Scholar, CAPES theses and dissertations library, Clinicaltrials and gray literature (SIGLE).

### Search

The search strategies were created using controlled vocabulary terms specific to each database and free terms relevant to the research question. These were sought in the titles and abstracts of the articles. No filters were used and there were no limitations related to the year of publication or language. Additionally, the search included a manual search of cross-references of the original articles and reviews to identify additional studies that were not identified in the databases. The search strategy used in each database, the search date and the number of retrieved studies is shown in Supplementary Table S1.

### Study selection

The references were taken to the Rayyan QCRI application, a tool used for the screening process of titles and abstracts according to the inclusion criteria (PICOS). After removing duplicate records of the same report, two reviewers carried out the selection process by reading the titles and abstracts independently to include articles for full reading. The full text of every potentially relevant study was then obtained to determine its eligibility for inclusion. The reasons for exclusion were recorded. When there was no consensus between the two reviewers regarding the inclusion of an article, a third researcher was consulted to analyze the divergences.

### Data collection process and data items

The data were extracted using a standardized form designed for this review in the Excel software. Two reviewers performed the data extraction independently, and consensus resolved disagreements. If necessary, a third reviewer resolved disagreements. The following data were extracted: publication data (author, year of publication, country and language); characteristics of the studies (study design, setting, size of each treatment arm, study duration, conflict of interest and funding); characteristics of the participants (type of teeth, age of participants, criteria used, type of study, gender, age,); characteristics of the intervention (route of administration, frequency of treatment, adjuvant therapy); evaluated outcome (secondary caries lesions) and the respective time points.

### Geometry of the network

Whenever possible, network meta-analyses (NMA) were performed, grouped by teeth, permanent and deciduous, and by the evaluated follow-up moments, 01 year or 2 or more years. Interventions through restorations with bioactive or conventional materials represent nodes. The group of GIC included high-viscosity glass ionomer (HVGIC) and conventional glass ionomer (CIG) in a single node. The authors made this decision because the control of carious lesions with glass ionomer cement is primarily attributed to fluoride release, regardless of viscosity. Both glass ionomer cements, conventional and high-viscosity, have fluorine-releasing and recharge properties similar.

### Risk of bias within individual studies

The risk of bias in studies with RCT design was assessed at the outcome level using the Cochrane Risk of Bias 2.0 (RoB 2) tool, and o non-randomized studies using the Risk of Bias in Non-randomized Studies—of Interventions (ROBINS-I) tool [22, 23]. Two independent reviewers performed this assessment with subsequent consensus. In evaluating RCTs using the RoB 2 tool, the reviewer assessed the following domains: bias in the randomization process, bias due to deviations from intended interventions, bias due to missing outcome data, bias in outcome measurement, and bias in the selection of reported results. RCTs were judged for each outcome as low risk of bias (if all domains were judged as low risk), some concerns (if at least one domain was judged as some concerns and no domain judged as high risk), and high risk of bias (if at least one domain was judged as high risk or if multiple domains were judged as some concerns).

In the evaluation of non-randomized studies using the ROBINS-I tool, seven domains were assessed: bias due to confounding, bias due to participant selection, bias in classification of interventions, bias due to deviations from intended interventions, bias due to missing data, bias in outcome measurement, and bias in the selection of reported results. The domains were judged as critical, serious, moderate, low, or no information. Non-randomized studies received an overall judgment for each assessed outcome. They were judged as low risk of bias if all domains were classified as low risk, moderate risk of bias if there was a low or moderate judgment for all domains, serious risk of bias if there was a serious risk of bias judgment in at least one domain, critical risk of bias if at least one domain was classified as critical, and no information if there is no clear indication that the study is at serious or critical risk of bias and there is a lack of information in one or more key domains of bias.

### Summary measures and planned methods of analysis

A meta-analysis was performed for each pair of compared intervention when sufficient homogeneous data was available. When two publications of the same study were identified, the data from the most recent study were used. The analyses were obtained using the random effects model to calculate the combined effect treatment and the respective 95% CI. These were grouped by dentition, permanent and deciduous, as well as by the evaluated moments of follow-up, 01 year or 2 or more years. Paired meta-analyses were performed using the Review Manager software, v. 5.3. When possible, network meta-analyses were conducted with the random effects model and a frequentist approach to estimate the relative effects for all possible comparisons between any pair of treatments at different moments of follow-up. The network meta-analyses were performed in NMAstudio [24], a web application for producing and visualizing interactive results of network meta-analyses. The heterogeneity assessment in each paired comparison was performed by visually inspecting the similarity of point estimates and the overlapping of confidence intervals and using the Chi^2^ test and I^2^ measure. The evaluation of the statistical heterogeneity of the complete network was based on Tau^2^.

### Risk of bias across studies

Analysis of publication bias was made by visually inspecting the funnel plot when more than ten studies were included.

### Additional analyses

Sensitivity analyses were performed for primary outcomes when sufficient data were available, considering the risk of bias (RCTs with low risk of bias).

### Certainty assessment

The certainty of evidence was assessed for each primary outcome in each comparison using the GRADE approach. The overall quality of the evidence was classified as high, moderate, low, or very low.

## Results

### Study selection

**Fig. 1:**
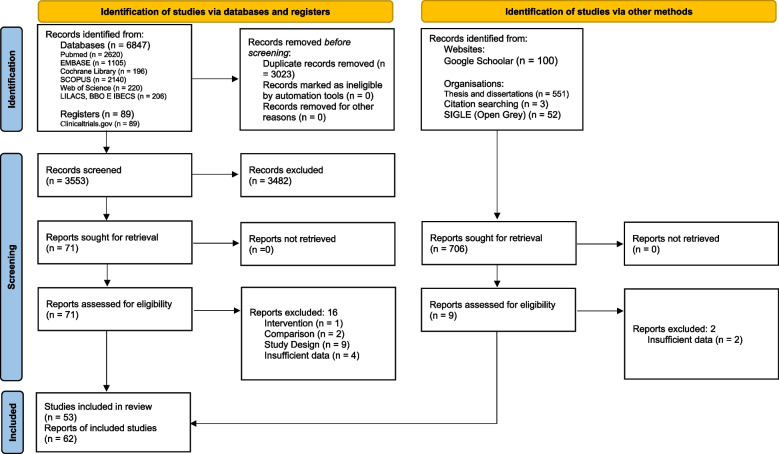
PRISMA flowchart of study selection

Initially, 6,487 articles were identified in the databases, 89 from records and 706 from the gray literature. After removing the duplicates and screening through titles and abstracts, 80 references were selected for reading in full and possible confirmation of eligibility. Of these, 18 were excluded for not meeting the inclusion criteria and the reasons for exclusions are presented in Supplementary Table S2. In the end, 62 reports of studies were included, all analyzed qualitatively. However, of the 62, only 39 presented sufficient data for quantitative synthesis (meta-analysis). The study selection process is described in Fig. 1.

### Presentation of network structure and summary of network geometry

**Fig. 2:**
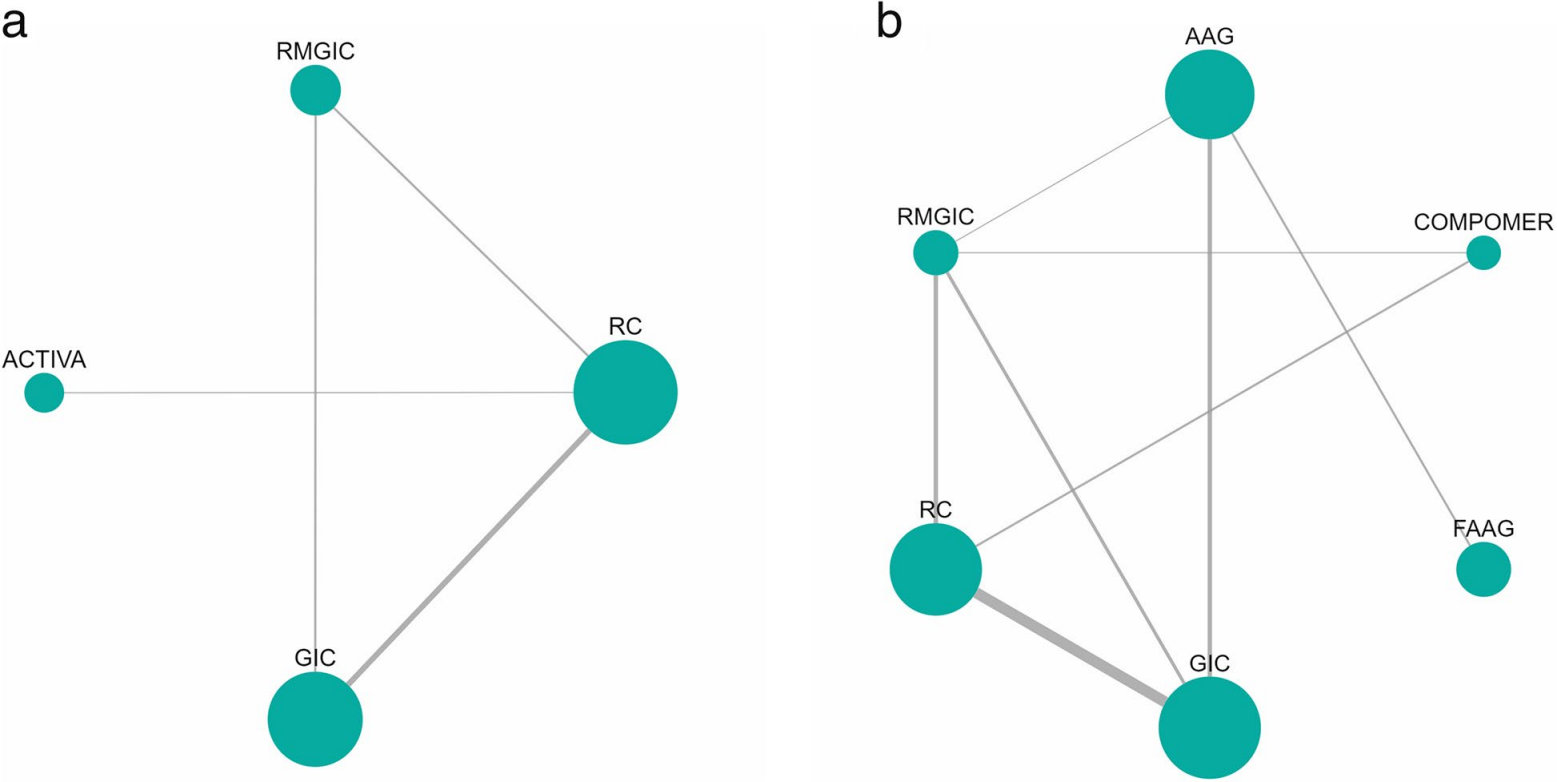
Analysis of restorative materials for the SC outcome in permanent teeth for 1 year and 2 or more years of follow-up: **a** Network diagram for 1 year of follow-up; **b** Network diagram for a follow-up of 2 years or more. *Abbreviations:* AAG: Amalgam; GIC: Glass Ionomer Cement; RMGIC: Resin Modified Glass Ionomer Cement; FAAG: Fluoride-containing Amalgam; RC: Resin Composite

The network diagrams for the SC outcome in permanent teeth, for a follow-up of 01 year and 02/more years are depicted in Fig. 2a and b, respectively. Six studies were included in the network meta-analysis that evaluated SC in permanent teeth at a follow-up time of 01 year and eighteen studies in the network meta-analysis that evaluated SC at a follow-up time of 02/more years. The size of the nodes is proportional to the total number of participants allocated to each intervention.

**Fig. 3:**
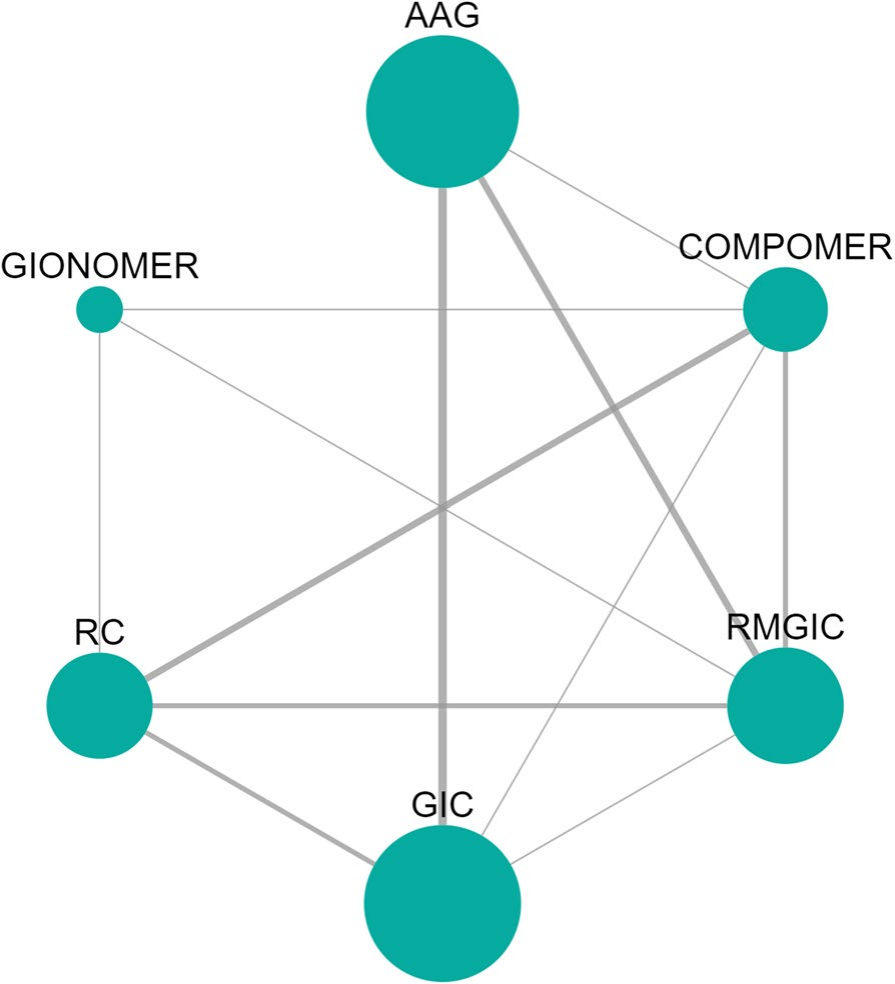
Network meta-analysis estimates of interventions *versus* GIC for the SC outcome in deciduous teeth with a follow-up of 02/more years. *Abbreviations:* AAG: Amalgam; GIC: Glass Ionomer Cement; RMGIC: Resin Modified Glass Ionomer Cement; RC: Resin Composite

The network diagram for the SC outcome in deciduous teeth, for the follow-up time of 02/more years is shown in Fig. 3. Fourteen studies were included in the network meta-analysis that evaluated SC in deciduous teeth at a follow-up time of 02/more years.

### Study characteristics and results of individual studies

Regarding the design, the 62 articles included in the review comprised randomized clinical trials [14, 25–37], controlled [14, 25, 34, 38, 39], split-mouth [28, 35, 36, 39–63], parallel [30, 32, 33, 64–70], only non-randomized and non-controlled clinical trial [71, 72], retrospective clinical [73] and some articles did not specify their design [74–80]. The results of individual studies are presented in Supplementary Table S3.

For the analysis of the occurrence of SC, the studies applied, in the great majority, the USPHS criterion [26, 28, 29, 32–38, 42, 44, 46–55, 58–62, 67–69, 73, 75]; the FDI [31, 63], Cvar and Ryge criteria [25, 45, 46, 56], Pitts 1984 [39], Frencken for Atraumatic Restorative Treatment (ART) [25, 68, 70], Mc Comb 2002 [81], Duperon et al.,1994 [57], adapted from Wood et al. 1993 [72], and ART criterion [30, 32, 33, 64, 68] were also used. Some articles did not report the criteria used. [14, 29, 39, 43, 48, 50, 53, 58, 60, 66, 71, 74].

Regarding the isolation of the operative field, absolute isolation [14, 25, 26, 28, 29, 35, 37–39, 48, 50, 53, 58–60, 65, 71, 72, 75, 79, 80, 82] or relative isolation [27, 30, 31, 34, 36, 42, 43, 45–47, 49, 52, 55–57, 61, 62, 66, 68–70, 76, 78, 83–85] was used, in both the intervention and control materials. The same study showed the use of absolute isolation in one of the evaluated restorative materials and relative isolation in another [32, 33, 59, 63], whereas some articles did not report the type of isolation used in the clinical study [40, 41, 44, 51, 54, 64, 67, 73, 74, 77, 81].

### Risk of bias within studies

**Fig. 4:**
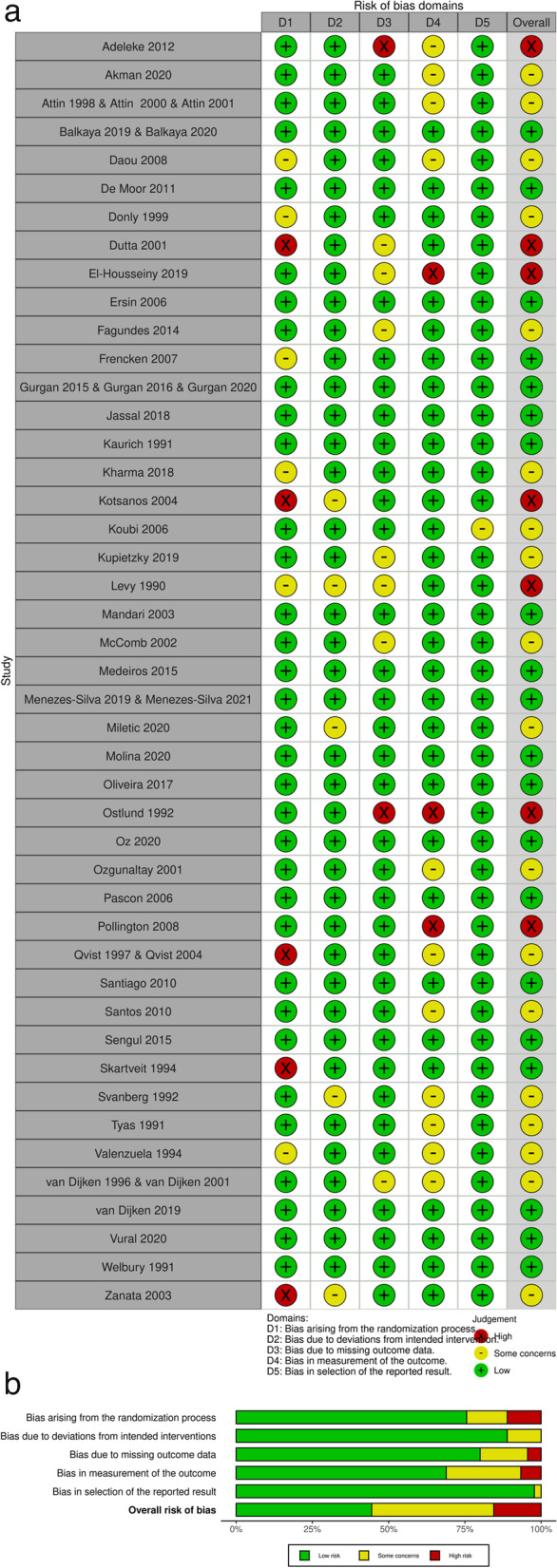
Risk of Bias of Randomized Clinical Trials: **a** Individual analysis of the articles; **b** Graph showing the abstracts of all articles

**Fig. 5:**
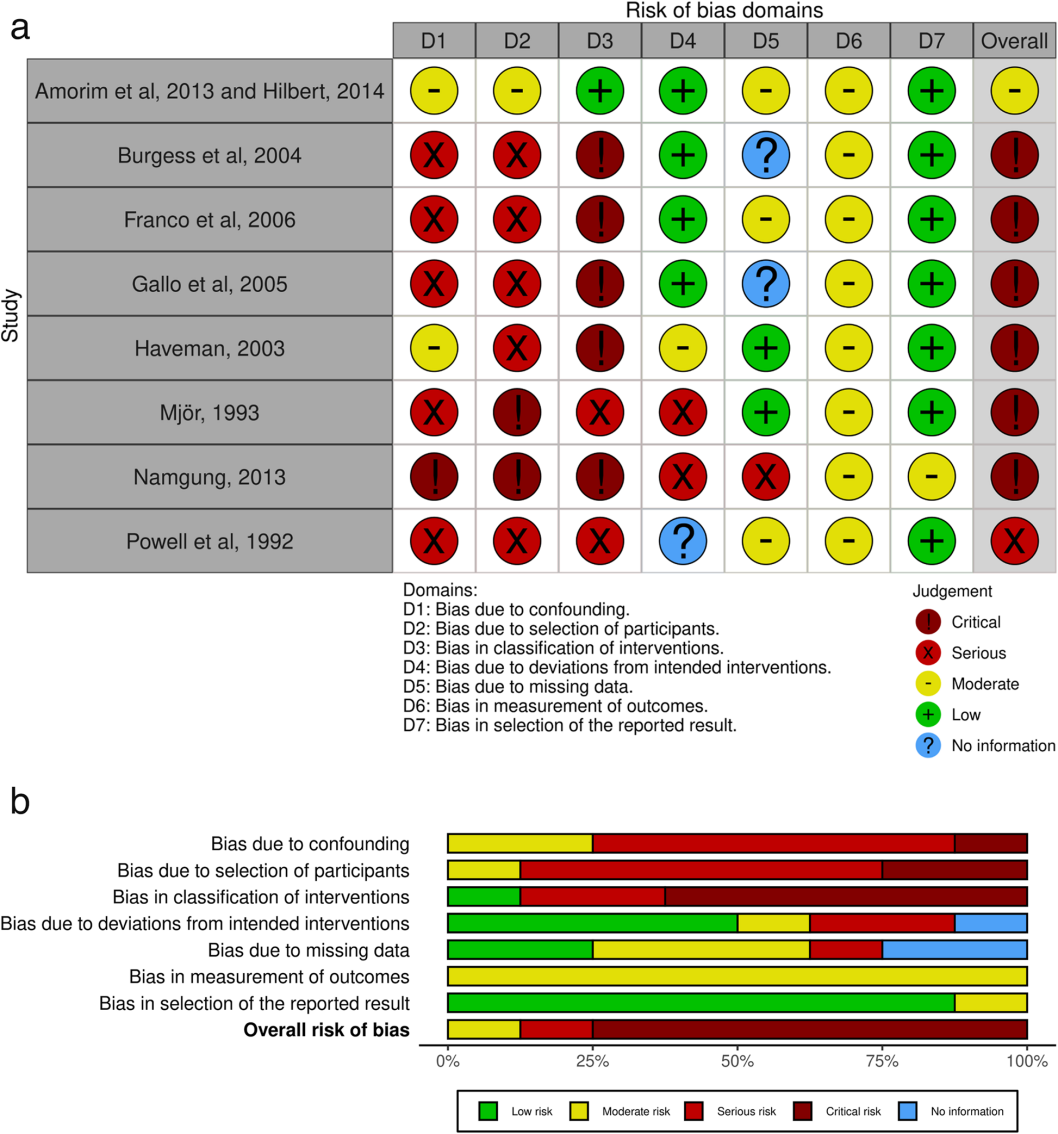
Risk of Bias of non-randomized studies assessed using the Risk of Bias in Non-randomized Studies—of Interventions (ROBINS-I) tool: **a** individual analysis of the articles; **b** graph showing the abstracts of all articles

The risk of bias at the primary outcome level was assessed considering the different comparisons and follow-up. The risk of bias in studies with RCT design is presented in Fig. 4 and the risk of bias in studies with non-randomized design is presented in Fig. 5. All randomized Clinical Trials showed more than 50% of low risk of bias in all evaluated criteria (Fig. 4a and b).

### Synthesis of results

#### Analysis in permanent teeth

The results of the network and paired meta-analyses, with the respective relative risks (RRs), are shown in the Table 1.

**Table 1:** Relative effects of the intervention as estimated from the network meta-analysis model, with 1 (a) and 2/more (b) years of follow-up. The lower triangle shows the result of the network meta-analysis and the upper triangle shows the result of the paired meta-analysis

a. 1 year				b. 2/more year					
**ACTIVA**		7.47 (0.36,154.12)		**AAG**		1.88 (1.14, 3.10)	1.70 (0.97, 2.97)		1.14 (0.43, 3.05)
22.80 (0.89,584.13)	**GIC**	0.33 (0.10, 1.06)	0.39 (0.08, 1.98)	0.56 (0.10, 3.21)	**COMPOMER**			1.56 (0.26, 9.37)	2.52 (0.26,24.20)
7.47 (0.36,154.12)	0.33 (0.10, 1.05)	**RC**	1.85 (0.68, 5.06)	1.88 (1.14, 3.10)	3.37 (0.55,20.72)	**FAAG**			
12.56 (0.52,303.50)	0.55 (0.14, 2.21)	1.68 (0.62, 4.53)	**RMGIC**	1.79 (1.04, 3.09)	3.20 (0.59,17.33)	0.95 (0.45, 2.00)	**GIC**	0.45 (0.23, 0.87)	0.92 (0.43, 1.98)
				0.89 (0.42, 1.92)	1.60 (0.32, 8.05)	0.47 (0.19, 1.19)	0.50 (0.28, 0.91)	**RC**	1.36 (0.64, 2.92)
				1.34 (0.64, 2.83)	2.40 (0.46,12.61)	0.71 (0.29, 1.75)	0.75 (0.39, 1.43)	1.50 (0.79, 2.85)	**RMGIC**

RRs < 1 favor treatment on the left and > 1 favor treatment on the right

**Fig. 6:**
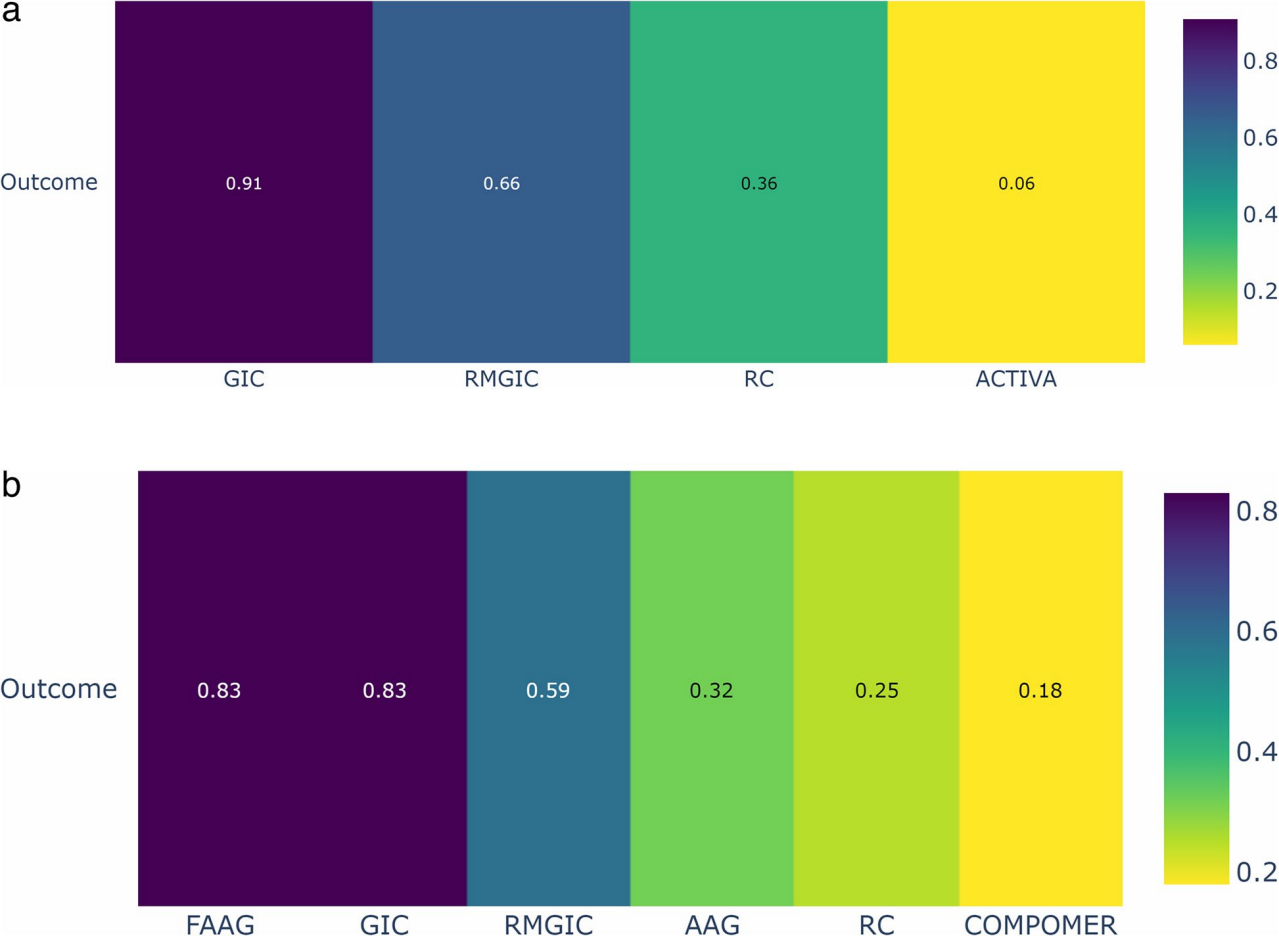
Analysis of restorative materials for the SC outcome in permanent teeth for 1 year and 2 or more years of follow-up: **a** Ranking of interventions for a follow-up of 1 year; **b** Ranking of interventions for a follow-up of 02 years or more. *Abbreviations:* AAG: Amalgam; GIC: Glass Ionomer Cement; RMGIC: Resin Modified Glass Ionomer Cement; FAAG: Fluoride-containing Amalgam; RC: Resin Composite

Based on the ranking (P-score) of the interventions according to Fig. 6a and b, it can be observed that the GIC group (one year of follow-up) and the GIC and Fluoride-containing Amalgam (FAAG) groups (follow-up of two years or more) had the highest probability of a positive response and a lower frequency of SC in permanent teeth, when compared to other restorative materials.

**Fig. 7:**
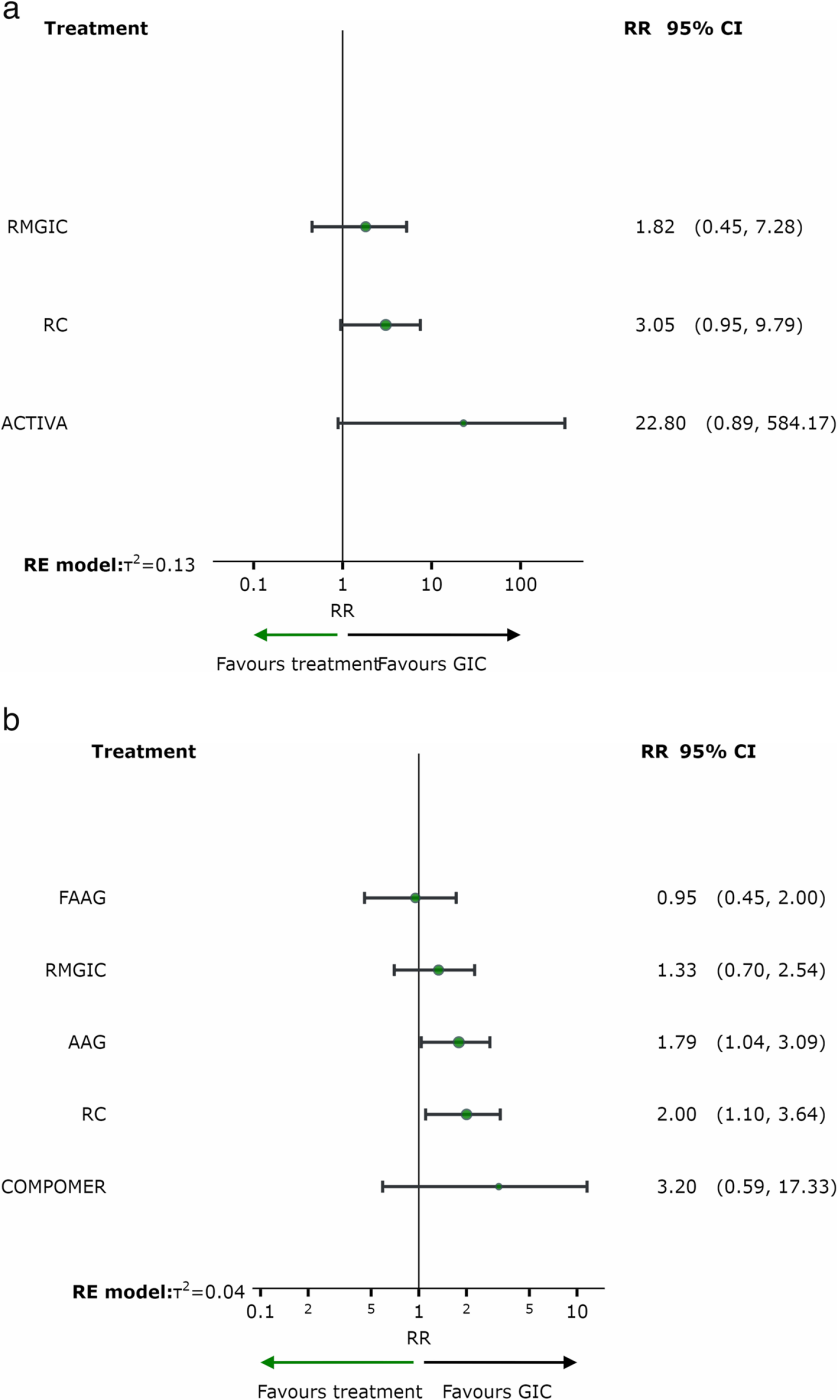
Network meta-analysis estimates of interventions *versus* GIC for the outcome of secondary caries in permanent teeth with a follow-up of 01 year (**a**) and 02/more years (**b**). *Abbreviations:* AAG: Amalgam; GIC: Glass Ionomer Cement; RMGIC: Resin Modified Glass Ionomer Cement; FAAG: Fluoride-containing Amalgam; RC: Resin Composite

Figure 7 shows the relative effects of network meta-analyses against GIC with their 95% confidence intervals. GIC was chosen as the reference because it was the material with the best ranking performance in both follow-up time periods.

After one year of follow-up, there was no difference between the materials in the occurrence of SC in permanent teeth, as shown in Fig. 7a. After two or more years of follow-up, the risk of occurrence of SC was significantly higher in the resin composite group than in the GIC group (RR = 2.00; 95% CI = 1.10, 3.64) and in the AAG group compared to the GIC group (RR = 1.79; 95% CI = 1.04, 3.09), as shown in Fig. 7b.

### Analysis in deciduous teeth

**Fig. 8:**
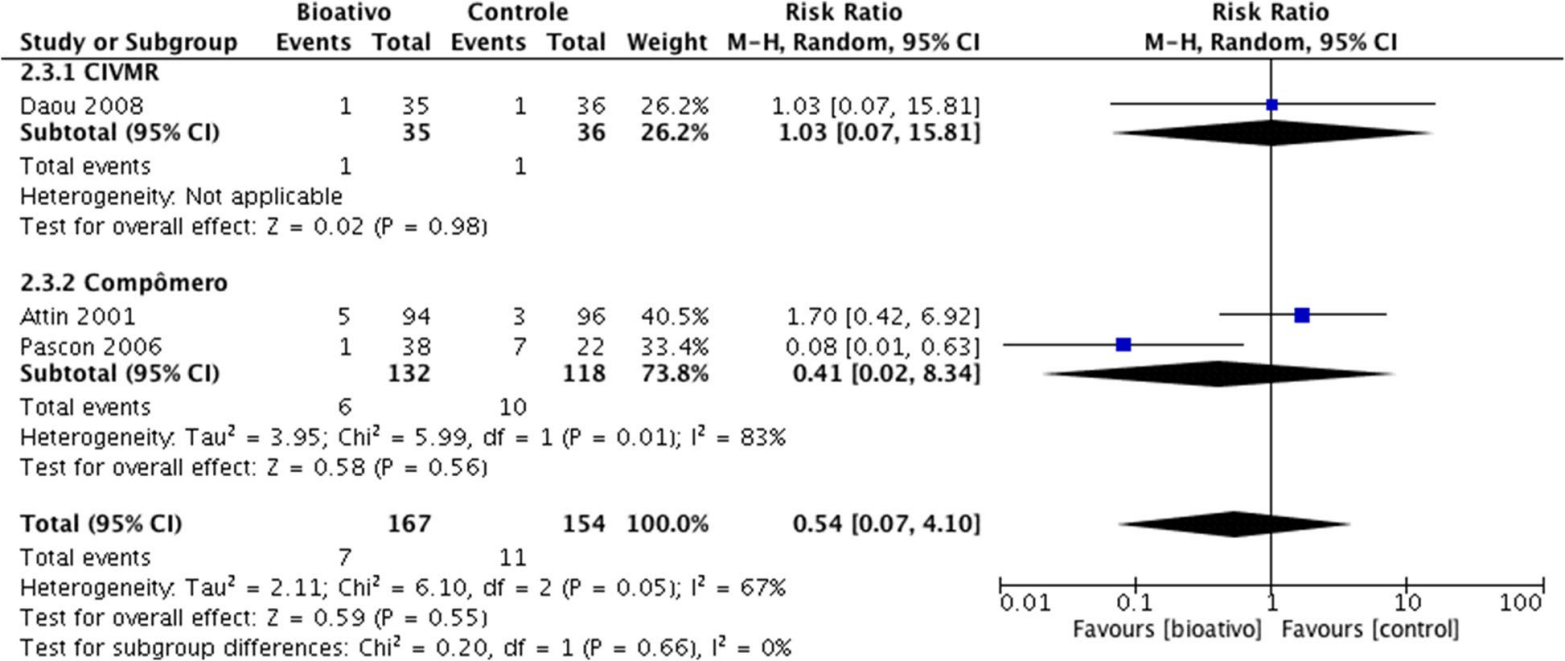
Analysis of restorative materials for the SC outcome in deciduous teeth: Paired meta-analysis estimates between bioactive materials and resin composite for a follow-up of 1 year. *Abbreviation:* CIVMR: Resin Modified Glass Ionomer Cement

After a follow-up of one year, it was not possible to perform a network meta-analysis, due to the few comparisons found. For this period of follow-up, we performed a paired meta-analysis, as shown in Fig. 8, with the resin composite as control. After one year of follow-up, there was no difference between the materials in relation to the occurrence of SC in deciduous teeth, as shown in Fig. 8. After two or more years of follow-up, the risk of SC was significantly higher in the resin composite group than in the AAg group (RR = 2.46; 95%CI = 1.42, 4.27) and in the GIC group compared to the RMGIC group (RR = 1.79; 95% CI = 1.04, 3.09), as shown in Fig. 7b.

The results of the network and paired meta-analyses, with the respective relative risks (RRs), are shown in the Table 2.

**Table 2:** Relative effects of the intervention as estimated from the network meta-analysis model. The lower triangle shows the result of the network meta-analysis and the upper triangle shows the result of the paired meta-analysis

AAG	0.33 (0.04, 3.06)	1.25 (0.71, 2.17)			2.42 (1.28, 4.58)
1.46 (0.75,2.85)	COMPOMER	0.69 (0.17, 2.85)	1.32 (0.38, 4.53)	1.06 (0.64, 1.77)	1.77 (0.79, 3.97)
1.28 (0.78,2.11)	0.88 (0.50,1.54)	GIC		1.17 (0.76, 1.81)	4.00 (0.47,33.91)
1.48 (0.43,5.13)	1.01 (0.33,3.15)	1.15 (0.35,3.84)	GIONOMER	2.10 (0.41,10.84)	1.69 (0.33, 8.66)
1.54 (0.86,2.76)	1.05 (0.66,1.69)	1.20 (0.81,1.78)	1.04 (0.32,3.33)	RC	1.12 (0.43, 2.93)
2.46 (1.42,4.27)	1.68 (0.87,3.25)	1.92 (1.03,3.56)	1.66 (0.49,5.61)	1.60 (0.85,3.00)	RMGIC

RRs < 1 favor treatment on the left and > 1 favor treatment on the right

**Fig. 9:**
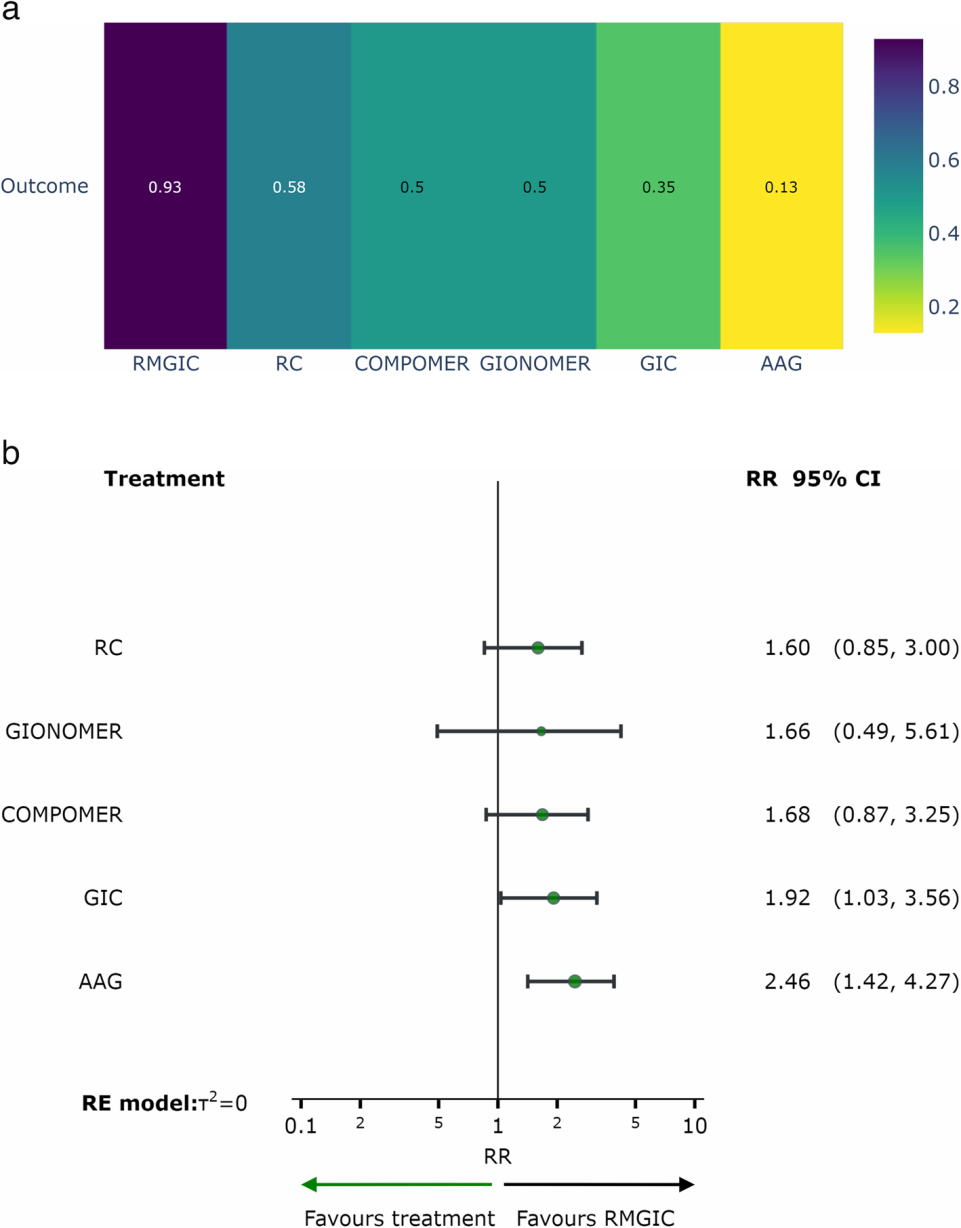
Analysis of restorative materials for the SC outcome in deciduous teeth with a follow-up of 02/more years: **a** Ranking of interventions for a follow-up of 02/more years; **b** Network meta-analysis estimates of interventions *versus* RMGIC for a follow-up of 02/more years. *Abbreviations:* AAG: Amalgam; GIC: Glass Ionomer Cement; RMGIC: Resin Modified Glass Ionomer Cement; RC: Resin Composite

Based on the ranking (P-score) of the interventions shown in Fig. 9a, it was observed that the RMGIC group (follow-up of two/more years) had the highest probability of a positive response, with a lower frequency of SC in deciduous teeth, when compared to other restorative materials. Figure 9b shows the relative effects of the network meta-analyses against Resin Modified Glass Ionomer Cement, with the 95% confidence intervals. RMGIC was chosen as the reference because it was the material with the best ranking performance.

### Risk of bias across studies

**Fig. 10:**
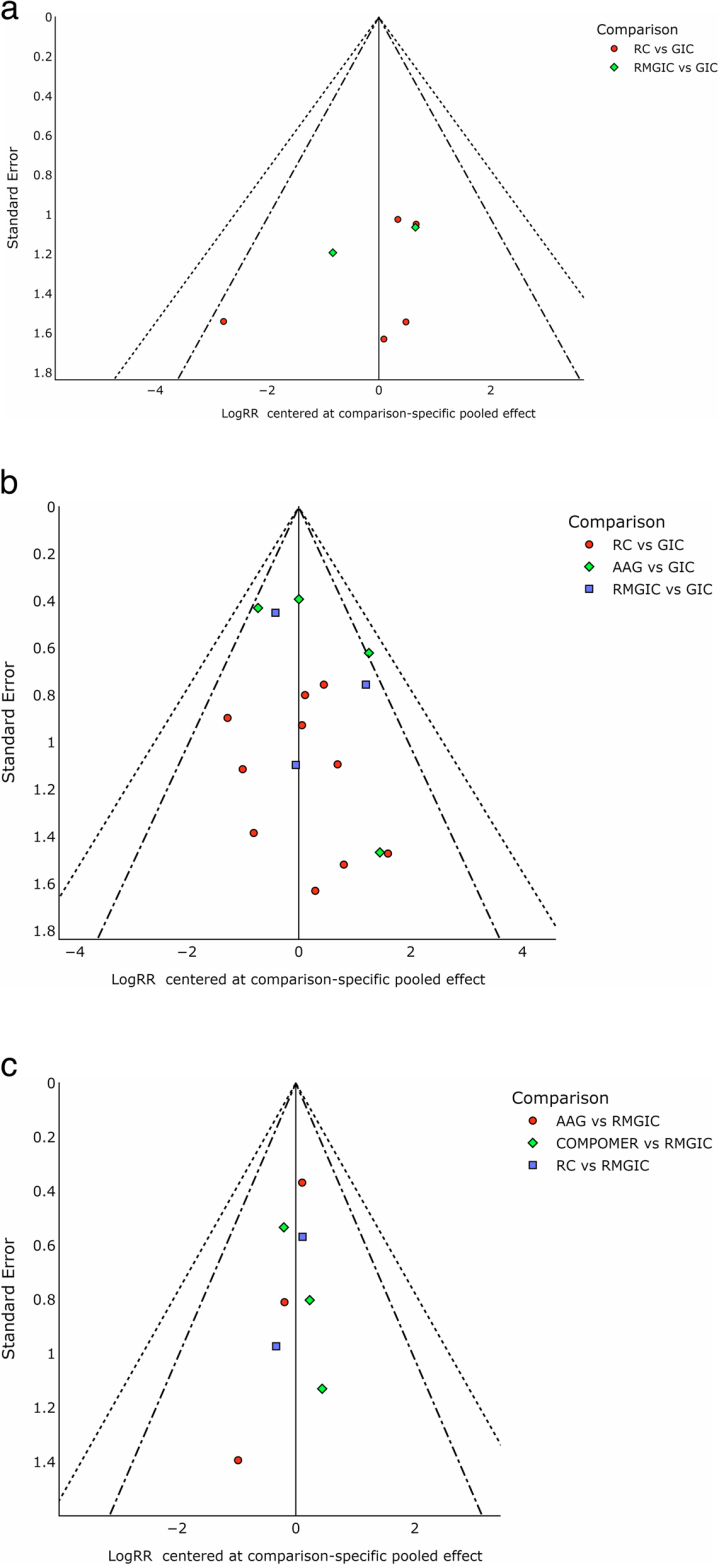
Funnel plots for the network meta-analysis of all primary outcomes in clinical trials – **a** Permanent teeth follow-up of 1 year; **b** Permanent teeth follow-up of 2 years/more; **c** Deciduous teeth follow-up of 2 years/more

The publication bias was evaluated considering the comprehensive search strategies performed and observed through visual inspection of the funnel plot (Fig. 10), when more than 10 studies were included, evaluating the same follow-up duration. For the one-year follow-up in deciduous teeth, the risk of publication bias was assessed considering only comprehensive search strategies, due to the limited number of included studies. The probability of unpublished studies was considered to be low.

### Results of additional analyses

**Fig. 11:**
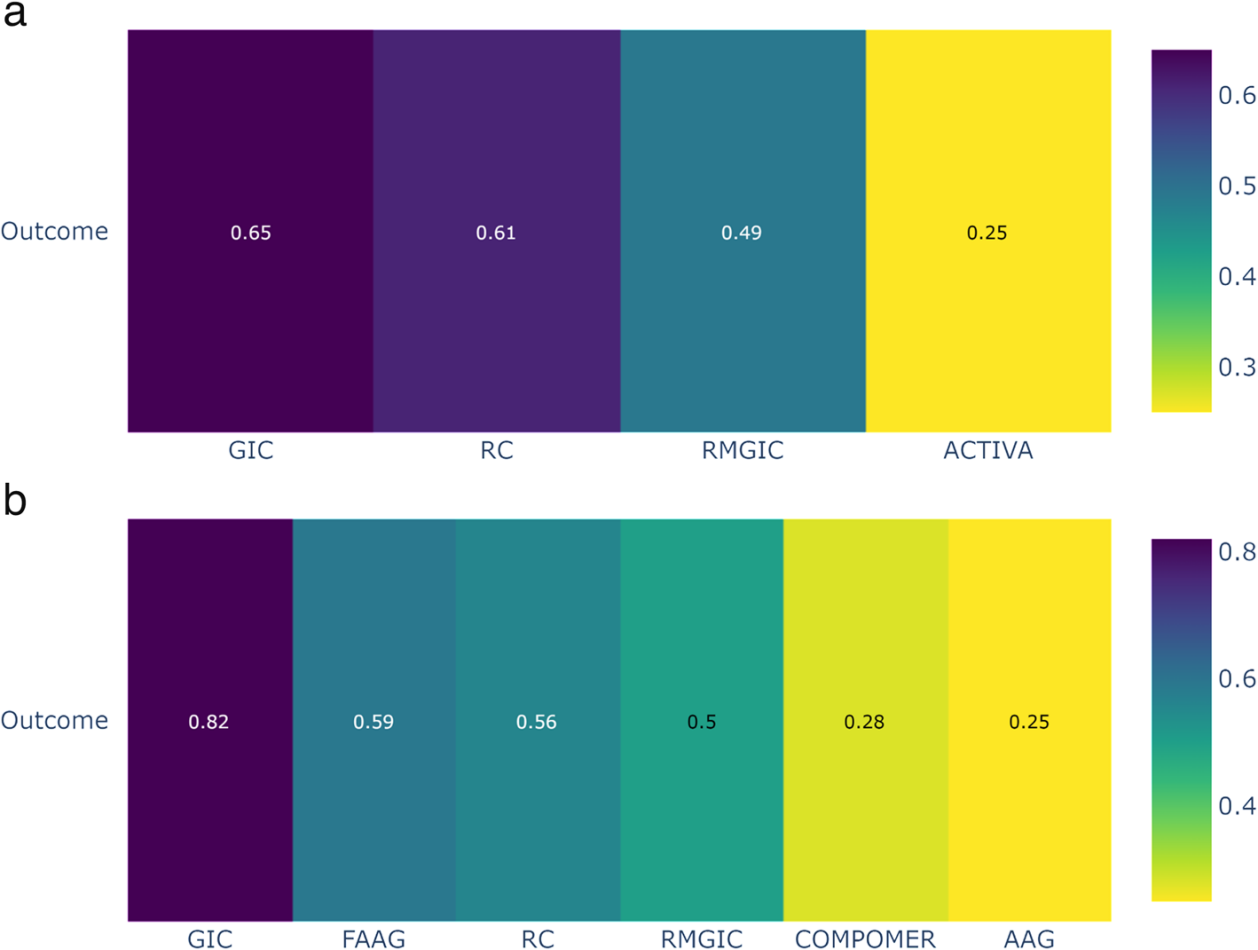
Sensitivity analyses were performed including only RCTs with low risk of bias. Analysis of restorative materials for the SC outcome in permanent teeth for 1 year and 2 or more years of follow-up: **a** Ranking of interventions for a follow-up of 1 year; **b** Ranking of interventions for a follow-up of 02 years or more. *Abbreviations:* AAG: Amalgam; GIC: Glass Ionomer Cement; RMGIC: Resin Modified Glass Ionomer Cement; FAAG: Fluoride-containing Amalgam; RC: Resin Composite

We conducted sensitivity analyses that included only randomized controlled trials (RCTs) with a low risk of bias. The network meta-analysis (NMA) for the outcome of secondary caries in permanent teeth at a one-year follow-up, which included RCTs with a low risk of bias, showed similar results to the main analysis. However, Resin Composite and Resin Modified Glass Ionomer Cement changed their rankings in the comparison (Fig. 11a). In the NMA for the outcome of secondary caries in permanent teeth with a follow-up of two or more years, which also included RCTs with a low risk of bias, Glass Ionomer Cement is also the superior intervention. However, the rankings of all the other materials were different (Fig. 11b).

**Fig. 12:**
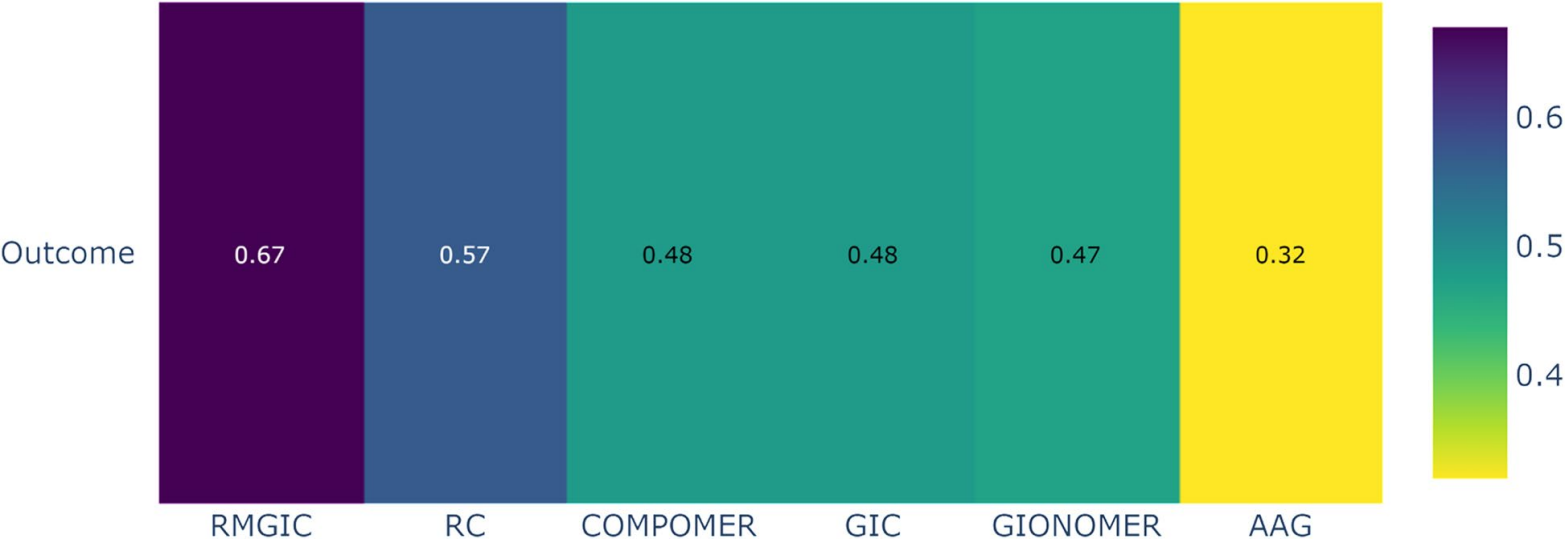
Ranking of restorative materials for the SC outcome in deciduous teeth with a follow-up of 02/more years. *Abbreviations:* AAG: Amalgam; GIC: Glass Ionomer Cement; RMGIC: Resin Modified Glass Ionomer Cement; RC: Resin Composite

The network meta-analysis (NMA) for the outcome of secondary caries in deciduous teeth with a follow-up of 02/more years, which included RCTs with a low risk of bias, showed similar results to the main analysis. However, GIOMER and Glass Ionomer Cement changed their positions in the ranking (Fig. 12).

### Certainty in the evidence

The assessment of the certainty of evidence regarding the occurrence of secondary caries in permanent teeth after a one-year follow-up varied across all analyzed comparisons, ranging from very low to low (Table 3). While in the follow-up of 2/more years, it ranged from very low to moderate confidence levels (Table 4).

**Table 3:** Summary of findings: SC outcome in permanent teeth for 1 year of follow-up

Secondary Caries outcome in permanent teeth for 1 year of follow-up					
**Patient or population:** Permanent teeth **Interventions:** ACTIVA; GIC: Glass Ionomer Cement; RMGIC: Resin Modified Glass Ionomer Cement; RC: Resin Composite					
Interventions	**Anticipated absolute effects** **with intervention**	Relative effect (95% CI)	№ of participants (studies)	Certainty of the evidence (GRADE)	Ranking
GIC	1 per 100	**RR 0.33**^d^ (0.10 to 1.05)	380 (5 Clinical Trials)	⨁⨁◯◯ Low^a,b^	1º—P-score = 0.91
RMGIC	8 per 100	**RR 1.82**^e^ (0.45 to 7.28)	126 (2 Clinical Trials)	⨁⨁◯◯ Low^a,b^	2º—P-score = 0.66
RC	3 per 100	**RR 3.05**^e^ (0.95 to 9.79)	479 (6 Clinical Trials)	⨁⨁◯◯ Low^a,b^	3º—P-score = 0.36
ACTIVA	4 per 100	**RR 22.80**^e^ (0.89 to 584.17)	153 (1 Clinical Trial)	⨁◯◯◯ Very low^c^	4º—P-score = 0.06

^a^One-level reduction due to some concerns regarding the risk of bias in the primary studies

^b^One-level reduction due to imprecision

^c^Significant concerns regarding the imprecision of the estimates

^d^The relative effect is based on comparison with RC

^e^The relative effect is based on comparison with GIC

**Table 4:** Summary of findings: outcome in permanent teeth for 2 or more years of follow-up

Secondary Caries outcome in permanent teeth for 2 or more years of follow-up					
**Patient or population:** Permanent teeth **Interventions:** GIC: Glass Ionomer Cement; RMGIC: Resin Modified Glass Ionomer Cement; RC: Resin Composite; AAG:Amalgam; FAAG: Fluoride-containing Amalgam; COMPOMER					
Interventions	**Anticipated absolute effects** **with intervention**	Relative effect (95% CI)	№ of participants (studies)	Certainty of the evidence (GRADE)	Ranking
GIC	2 per 100	**RR 0.50**^d^ (0.28 to 0.91)	1571 (14 Clinical Trials)	⨁⨁⨁◯ Moderate^a^	1º—P-score = 0.83
FAAG	16 per 100	**RR 0.95**^e^ (0.45 to 2.00)	309 (6 Clinical Trials)	⨁⨁◯◯ Low^a,b^	2º—P-score = 0.83
RMGIC	11 per 100	**RR 1.33**^e^ (0.70 to 2.54)	141 (5 Clinical Trials)	⨁⨁◯◯ Low^a,b^	3º—P-score = 0.59
AAG	24 per 100	**RR 1.79**^e^ (1.04 to 3.09)	390 (7 Clinical Trials)	⨁⨁◯◯ Low^a,b^	4º—P-score = 0.32
RC	6 per 100	**RR 2.00**^e^ (1.10 to 3.64)	728 (13 Clinical Trials)	⨁◯◯◯ Very low^a,c^	5º—P-score = 0.25
COMPOMER	3 per 100	**RR 3.20**^e^ (0.59 to 17.33)	79 (2 Clinical Trials)	⨁◯◯◯ Very low^a,c^	1º—P-score = 0.18

^a^One-level reduction due to some concerns regarding the risk of bias in the primary studies

^b^One-level reduction due to imprecision

^c^Significant concerns regarding the imprecision of the estimates

^d^The relative effect is based on comparison with RC

^e^The relative effect is based on comparison with GIC

The assessment of the certainty of evidence regarding the occurrence of secondary caries outcome in deciduous teeth with a follow-up of 02/more years varied from low to moderate confidence levels (Table 5).

**Table 5:** Summary of findings: outcome in deciduous teeth with a follow-up of 02/more years

Secondary Caries outcome in deciduous teeth with a follow-up of 02/more years					
**Patient or population:** Deciduos teeth **Interventions:** AAG: Dental Amalgam; GIC: Glass Ionomer Cement; RMGIC: Resin Modified Glass Ionomer Cement; RC: Resin Composite; COMPOMER; GIOMER					
Interventions	**Anticipated absolute effects** **with intervention**	Relative effect (95% CI)	№ of participants (studies)	Certainty of the evidence (GRADE)	Ranking
RC	13 per 100	**RR 1.60**^c^ (0.85 to 3.00)	438 (8 Clinical Trials)	⨁⨁◯◯ Low^a,b^	2º—P-score = 0.58
COMPOMER	15 per 100	**RR 1.68**^c^ (0.87 to 3.25)	207 (5 Clinical Trials)	⨁⨁◯◯ Low^a,b^	3º—P-score = 0.50
GIONOMER	8 per 100	**RR 1.66**^c^ (0.49 to 5.61)	38 (1 Clinical Trial)	⨁⨁◯◯ Low^a,b^	4º—P-score = 0.50
GIC	5 per 100	**RR 1.92**^c^ (1.03 to 3.56)	1193 (7 Clinical Trials)	⨁⨁⨁◯ Moderate^a^	5º—P-score = 0.35
AAG	5 per 100	**RR 2.46**^c^ (1.42 to 4.27)	1135 (8 Clinical Trials)	⨁⨁⨁◯ Moderate^a^	1º—P-score = 0.13

^a^One-level reduction due to some concerns regarding the risk of bias in the primary studies

^b^One-level reduction due to imprecision

^c^The relative effect is based on comparison with RMGIC

## Discussion

Although it is plausible to consider the use of bioactive materials with the purpose of controlling the DEM-REM process, the isolated analysis of materials makes the clinical choice a difficult one. At the same time, the limitations of in vitro studies are relevant in terms of representing the complexity of the oral environment and other etiological factors of dental caries. Even in situ studies that seek to expose the substrate to an oral environment may overestimate the rates of progression of caries lesions [86]. Therefore, the present study sought to compile the existing bioactive restorative materials on the market, containing fluoride, calcium and/or phosphate, aiming at a critical analysis of methodological issues found in the clinical trials published to date.

The ranking of restorative materials used in clinical practice showed a better performance of GIC for the control of SC in permanent teeth, whereas for deciduous teeth, the best performance for the same outcome was demonstrated by the RMGIC. Therefore, among the bioactive materials, those with greater release of F ions demonstrated greater efficacy in controlling SC. The other restorative materials, such as compomer, gionomer and Activa, do not have high fluoride release or even adequate fluoride recharge [87]. Fluoride release and recharge characteristics depend on the matrix, filler type, as well as fluoride content and type in the material [20]. In addition to the composition itself and the setting mechanism, the need to use an adhesive system prevents the passage of ions from the restorative material to the tooth structure, making the REM process difficult [87]. This justifies the inferior clinical result for the SC outcome of compomer, gionomer and Activa, when compared to conventional restorative materials such as amalgam and resin composite [25, 35, 36, 54, 56, 77].

When comparing restorative techniques with bioactive and conventional materials in the control of dental DEM, it was observed that the good oral hygiene of selected patients is a factor that can influence the results regarding the development of SC [60], as well as biofilm control, advice on diet and exposure to fluoride performed during patient follow-up in clinical evaluations [14, 54, 88]. This has gained theoretical-methodological support considering that, the absence of SC in posterior teeth, in RC and GIC restorations classes I and II, after different periods of clinical follow-up (1, 3, 4, 6 and 10 years) was attributed to the patients' good oral hygiene status [83]. Therefore, the risk of the formation of new lesions in these patients was considered low, regardless of the material used.

Regarding the specific protection of fluoride in individuals with a low or moderate risk of caries, the fluoride obtained from toothpaste and drinking water is sufficient to prevent the appearance of new lesions. In individuals at high risk of dental caries, the frequent decrease in pH hinders the action of sources with low concentration of fluoride, thus requiring additional sources, such as the restorative material itself [20, 54]. Bioactive materials can, therefore, effectively contribute to the control of the DEM-REM process, attenuating the progression of caries lesions, implying a reduction in the frequency of SC. Among the bioactive materials evaluated, GIC and RMGIC have advantages over RC, such as lower technical sensitivity and demand for less clinical time, due to the easy handling and insertion into the cavity [50]. Thus, they can be considered as first-choice materials, the GIC for permanent dentition and RMGIC for deciduous dentition, in high-risk and difficult-to-treat patients, such as non-collaborating children and special patients, as well as individuals living in regions with difficult access to oral health care, using the ART technique [65, 89].

The fact that the RMGIC is considered superior to the GIC in the deciduous dentition can be explained by the frequent GIC failures, especially in the proximal walls of the deciduous teeth. Access difficulty, small extension and lack of protection of the GIC can lead to porosity, as detected in clinical studies [69, 76] and the consequent loss of the proximal wall. GIC restorations showed defects with the aspect of concavities in the proximal wall [65], which may predispose to the development of caries lesions due to biofilm retention. Therefore, the technical difficulty and the low mechanical property of the GIC become more evident in deciduous teeth, with the use of RMGIC being preferable in this case.

The longevity of a restoration is multifactorial, as it depends on the handling, the operator, the adhesion capacity of the material, the way it is applied, in addition to factors related to the patient [49]. Although amalgam remains a restorative material of choice in different countries, given the current evidence and evolution of restorative materials, it is necessary to implement preventive procedures, as well as select and develop materials with less invasive approaches [90, 91]. Thus, although the amalgam with fluoride is ranked as one of the best materials for the control of SC in permanent teeth, at a follow-up of 2 years or more, this material was discontinued in the dental trade and is not currently available for clinical use. Also regarding the amalgam, especially in deciduous teeth, its use can lead to pulp exposure when the preparation principles are followed or even restoration loss, when these principles are not followed, making it difficult to retain the restoration. Therefore, its use in the deciduous dentition is not recommended.

Another point to be considered regarding clinical studies is the sample size. Usually, studies involving GIC and RMGIC showed the most representative number, which may impact the results, considering the variability found in patients. The fact that these materials have been on the market for a longer time than other bioactive restorative materials also influence the results, due to the greater number of clinical studies available in the literature. This can be observed in network analyses, where the nodes (circles) represent the sample size and the connections (lines) the number of studies. Although non-randomized clinical trials include more biases, as shown in Fig. 5, they generally have a more significant sample size and follow-up time, which is very interesting for the secondary caries outcome [92]. For this reason, the method of this study has not restricted the inclusion only to RCT.

SC was just one of the criteria observed in clinical studies and it can be observed that there is great difficulty for evaluators to attain a correct diagnosis and identification of the clinical characteristics of lesions at the margins of restorations. The presence of gaps without active lesions, discolorations and deterioration of the restoration margins can be mistakenly interpreted as early stages of SC, just as radiolucency in radiographic images may be indicative of residual caries or adhesive systems without radiopacity [93, 94]. Therefore, the studies may have a greater underreporting of the therapeutic effects of bioactive materials in relation to those that are not bioactive [71], in addition to promoting unnecessary restorative reinterventions. Another possible confounding factor is the criteria for failure that are not standardized among primary studies and might produce different results depending on the modifications used.

The selection of research participants can also influence the results obtained from SC cases [28, 69]. In well-standardized studies, which select patients without systemic diseases, with good oral hygiene, who collaborate and have no bruxism and clenching, there is a great chance of similarity between different materials regarding clinical longevity. As the vast majority of randomized controlled clinical trials disclose this selectivity, they often demonstrate a situation that is so controlled it does not correspond to reality [92]. This fact indicates the need for reflection on the interference of controlled experimental designs. One knows how important they are; however, it is necessary to think that they do not necessarily reflect the patient’s reality. Thus, results may be overestimated or underestimated, considering that the environment (oral cavity) and the experimental period differ from the reality experienced by the patient. Hence, it is worth considering that there may be consequences of controlled experimental procedures when these results are translated into clinical practice.

In some studies, a performance bias was observed by using different types of isolation of the operative field, both absolute and relative, between the control and comparison groups [32, 33, 59, 63], or by selecting the group that received oral health instruction and oral hygiene procedures [30]. That also happens with the selection bias, for a study where the allocation to the control group occurred in one school and the comparison group in another, therefore, without randomization [30, 64]. Few studies, as mentioned above, showed a risk of bias to be considered. The comprehensive search did not detect any publication bias. The most indicated material in each follow-up and type of evaluated tooth did not change with the sensitivity analysis, performed only with RCTs with a low risk of bias. This confirms that the construction of the network analysis was robust, and our findings are not statistical artifacts.

The quality of evidence in this NMA was moderate to low. The latter is more evident in the short evaluation time and small sample size. Therefore, primary studies should be multicentre with great samples and longer follow-ups. In addition, studies to evaluate the longevity of restorations should follow guidelines to standardize outcome reports to improve future comparisons.

Although there has been a large investment in the marketing of new products to control the DEM-REM process, preventing DEM and/or promoting REM, the restorative treatment alone does not control caries disease. It is necessary to identify the unbalanced risk factor exhibited by the patient and carry out an individualized control. However, the restorative material can be one more resource to be used in patients who are at high risk of dental caries.

## Conclusion

The authors concluded that there is a difference between the bioactive restorative materials to be indicated for SC control, with the GIC being the most effective in permanent teeth and the RMGIC showing the best performance, in this regard, for the restoration of deciduous teeth. The other evaluated bioactive restorative materials did not show superiority when compared to conventional materials (amalgam and resin composite), especially in the permanent dentition.

## Supplementary Information

**Additional file 1: Supplementary Table S1.** Search strategies performed in databases. **Supplementary Table S2.** List of excluded recordsin the full-text eligibility analysis. **Supplementary Table S3.** Characteristics as reported in the included clinical studies. All reports are described. When a single study was reported in more than one report, they were presented under the same Study ID.

## Data Availability

The datasets used and/or analysed during the current study available from the corresponding author on reasonable request.
