# Supplementary material for: Clinical efficacy of bioactive restorative materials in controlling secondary caries: a systematic review and network meta-analysis
Source: BMC Oral Health. 2023 Jun 15;23:394. doi: 10.1186/s12903-023-03110-y (PMC10268411; doi:10.1186/s12903-023-03110-y)
Supplement: Supplementary file 1 — Additional file 1: Supplementary Table S1. Search strategies performed in databases. Supplementary Table S2. List of excluded recordsin the full-text eligibility analysis. Supplementary Table S3. Characteristics as reported in the included clinical studies. All reports are described. When a single study was reported in more than one report, they were presented under the same Study ID. [file 12903_2023_3110_MOESM1_ESM.docx]

**Clinical efficacy of bioactive restorative materials in controlling secondary caries: a systematic review and network meta-analysis**

Noeleni Souza Pinto^1^, Gabriela Rebouças Jorge^1^, Jader Vasconcelos^2^, Livia Fernandes Probst^3^, Alessandro De Carli^1^, Andrea Freire^1^

^1^School of Dentistry, Universidade Federal de Mato Grosso do Sul, Campo Grande, Brazil

^2^ Secretaria de Saúde (SESAU), Campo Grande, Brazil.

^3^Unidade de Avaliação de Tecnologias em Saúde, Hospital Alemão Oswaldo Cruz, São Paulo, Brazil.

**Index of Supplementary Material**

This document contains an index of the supplementary material that accompanies our research paper. The supplementary material includes additional tables, figures, and data that were not included in the main paper due to space limitations.

The supplementary material is organized as follows:

[Supplementary Table S1. Search strategies performed in databases 2](#_Toc136450073)

[Supplementary Table S2. List of excluded records (and the reason for exclusion) in the full-text eligibility analysis 4](#_Toc136450074)

[Supplementary Table S3. Characteristics as reported in the included clinical studies. All reports are described. When a single study was reported in more than one report, they were presented under the same Study ID 5](#_Toc136450075)

Supplementary Table S1. Search strategies performed in databases

| **Database** | **Search strategies*** | **Results** |
| --- | --- | --- |
| PubMed | #1 (molar[MeSH Terms] OR molars[MeSH Terms] OR premolar[MeSH Terms] OR premolars[MeSH Terms] OR bicuspid[MeSH Terms] OR bicuspids[MeSH Terms] OR “canine teeth”[MeSH Terms] OR “canine tooth”[MeSH Terms] OR incisor[MeSH Terms] OR incisors[MeSH Terms] OR (tooth OR teeth OR dentin OR dentine OR enamel OR dental OR molar OR molars OR premolar OR premolars OR bicuspid OR bicuspids OR canine OR canines OR incisor OR incisors)  #2 (“glass ionomer cement”[MeSH Terms] OR “glass ionomer cements”[MeSH Terms] OR fluoride[MeSH Terms] OR fluorine[MeSH Terms] OR glass[MeSH Terms] OR glasses[MeSH Terms] OR apatites[MeSH Terms] OR hydroxyapatite[MeSH Terms] OR hydroxyapatites[MeSH Terms] OR casein[MeSH Terms] OR ("glass ionomer cement" OR “glass ionomer cements" OR "glass-ionomer-cement" OR "glass-ionomer cement" OR "glass ionomer" OR fluoride OR fluorine OR glass OR glasses OR apatites OR hydroxyapatite OR hydroxyapatites OR casein OR calcium OR “ACP-CPP” OR “amorphous calcium phosphate” OR phosphate OR “surface pre-reacted glass ionomer” OR “glass carbomer” OR “glass carbomer cement” OR bioactive)  #3 (“composite resins”[MeSH Terms] OR “dental cement”[MeSH Terms] OR “dental cements”[MeSH Terms] OR adhesive [MeSH Terms] OR “dental amalgam”[MeSH Terms] OR “dental amalgams”[MeSH Terms] OR ("composite resin" OR“composite resins” OR “resin based-composite” OR “composite restoration” OR “composite restorations” OR “dental cement” OR “dental cements” OR “adhesive system” OR “adhesive systems” OR “self-adhesive” OR “self-etch adhesive” OR “total-etch adhesive” OR “dental amalgam” OR “dental amalgams”)  #4 (“tooth demineralization”[MeSH Terms] OR “tooth remineralization”[MeSH Terms] OR “dental caries”[MeSH Terms] OR “dental decay”[MeSH Terms] OR (demineralization OR demineralized OR remineralization OR remineralising OR remineralize OR "dental caries" OR "dental decay" OR “secondary caries")  #1 AND #2 AND #3 AND #4 | 2620 |
| Web of Science | #1 TS = (molar OR molars OR premolar OR premolars OR bicuspid OR bicuspids OR “canine teeth” OR “canine tooth” OR incisor OR incisors) OR TI = (tooth OR teeth OR dentin OR dentine OR enamel OR dental OR molar OR molars OR premolar OR premolars OR bicuspid OR bicuspids OR canine OR canines OR incisor OR incisors)  #2 TS = “glass ionomer cement” OR “glass ionomer cements” OR fluoride OR fluorine OR glass OR glasses OR apatites OR hydroxyapatite OR hydroxyapatites OR casein) OR TI = ("glass ionomer cement" OR “glass ionomer cements" OR "glass-ionomer-cement" OR "glass-ionomer cement" OR "glass ionomer" OR fluoride OR fluorine OR glass OR glasses OR apatites OR hydroxyapatite OR hydroxyapatites OR casein OR calcium OR “ACP-CPP” OR “amorphous calcium phosphate” OR phosphate OR “surface pre-reacted glass ionomer” OR “glass carbomer” OR “glass carbomer cement” OR bioactive)  #3 TS = (“composite resins” OR “dental cement” OR “dental cements” OR adhesive OR “dental amalgam” OR “dental amalgams”) OR TI = ("composite resin" OR“composite resins” OR “resin based-composite” OR “composite restoration” OR “composite restorations” OR “dental cement” OR “dental cements” OR “adhesive system” OR “adhesive systems” OR “self-adhesive” OR “self-etch adhesive” OR “total-etch adhesive” OR “dental amalgam” OR “dental amalgams”)  #4 TS = (“tooth demineralization” OR “tooth remineralization” OR “dental caries” OR “dental decay”) OR TI = (demineralization OR demineralized OR remineralization OR remineralising OR remineralize OR "dental caries" OR "dental decay" OR “secondary caries")  #1 AND #2 AND #3 AND #4 | 220 |
| The Cochrane Library | #1 molar OR molars OR premolar OR premolars OR bicuspid OR bicuspids OR “canine teeth” OR “canine tooth” OR incisor OR incisors  #2 “glass ionomer cement” OR “glass ionomer cements” OR fluoride OR fluorine OR glass OR glasses OR apatites OR hydroxyapatite OR hydroxyapatites OR casein OR calcium OR phosphate  #3 “composite resins” OR “dental cement” OR “dental cements” OR adhesive OR “dental amalgam” OR “dental amalgams”  #4 “tooth demineralization” OR “tooth remineralization” OR “dental caries” OR “dental decay”  #1 AND #2 AND #3 AND #4 | 196 |
| SCOPUS | #1 (TITLE-ABS-KEY (molar OR molars OR premolar OR premolars OR bicuspid OR bicuspids OR “canine teeth” OR “canine tooth” OR incisor OR incisors OR tooth OR teeth OR dentin OR dentine OR enamel OR dental))  #2 (TITLE-ABS-KEY (“glass ionomer cement” OR “glass ionomer cements” OR fluoride OR fluorine OR glass OR glasses OR apatites OR hydroxyapatite OR hydroxyapatites OR casein OR "glass-ionomer-cement" OR "glass-ionomer cement" OR "glass ionomer" OR calcium OR “ACP-CPP” OR “amorphous calcium phosphate” OR phosphate OR “surface pre-reacted glass ionomer” OR “glass carbomer” OR “glass carbomer cement” OR bioactive))  #3 (TITLE-ABS-KEY (“composite resins” OR “dental cement” OR “dental cements” OR adhesive OR “dental amalgam” OR “dental amalgams” OR "composite resin" OR “resin based-composite” OR “composite restoration” OR “composite restorations” OR “adhesive system” OR “adhesive systems” OR “self-adhesive” OR “self-etch adhesive” OR “total-etch adhesive”))  #4 (TITLE-ABS-KEY (“tooth demineralization” OR “tooth remineralization” OR “dental caries” OR “dental decay” OR "secondary caries" OR demineralised OR remineralization OR remineralising OR remineralize OR demineralization))  #1 AND #2 AND #3 AND #4 | 2140 |
| LILACS, BBO e IBECS (BVS) | #1 molar OR molars OR premolar OR premolars OR bicuspid OR bicuspids OR “canine teeth” OR “canine tooth” OR incisor OR incisors OR tooth OR teeth OR dentin OR dentine OR enamel OR dental  #2 “glass ionomer cement” OR “glass ionomer cements” OR fluoride OR fluorine OR glass OR glasses OR apatites OR hydroxyapatite OR hydroxyapatites OR casein OR "glass-ionomer-cement" OR "glass-ionomer cement" OR "glass ionomer" OR calcium OR “ACP-CPP” OR “amorphous calcium phosphate” OR phosphate OR “surface pre-reacted glass ionomer” OR “glass carbomer” OR “glass carbomer cement” OR bioactive  #3 “composite resins” OR “dental cement” OR “dental cements” OR adhesive OR “dental amalgam” OR “dental amalgams” OR "composite resin" OR “resin based-composite” OR “composite restoration” OR “composite restorations” OR “adhesive system” OR “adhesive systems” OR “self-adhesive” OR “self-etch adhesive” OR “total-etch adhesive”  #4 “tooth demineralization” OR “tooth remineralization” OR “dental caries” OR “dental decay” OR demineralization OR demineralized OR remineralization OR remineralising OR remineralize OR "dental caries" OR "dental decay" OR “secondary caries"  #1 AND #2 AND #3 AND #4 | 206 |
| EMBASE | #1 'tooth'/exp OR 'molar tooth'/exp OR 'premolar tooth'/exp OR 'canine tooth'/exp OR 'incisor'/exp OR 'enamel'/exp OR 'dentin'/exp  #2 'glass ionomer'/exp OR 'glass carbomer'/exp OR 'fluoride'/exp OR 'fluorine'/exp OR 'apatite'/exp OR 'hydroxyapatite'/exp OR 'casein'/exp OR 'phosphate'/exp OR 'calcium'/exp  #3 'resin'/exp OR 'tooth cement'/exp OR 'adhesive agent'/exp OR 'dental amalgam'/exp  #4 'demineralization'/exp OR 'remineralization'/exp OR 'dental caries'/exp  #1 AND #2 AND #3 AND #4  Query: ('tooth'/exp OR 'molar tooth'/exp OR 'premolar tooth'/exp OR 'canine tooth'/exp OR 'incisor'/exp OR 'enamel'/exp OR 'dentin'/exp) AND ('glass ionomer'/exp OR 'glass carbomer' OR 'fluoride'/exp OR 'fluorine'/exp OR 'apatite'/exp OR 'hydroxyapatite'/exp OR 'casein'/exp OR 'phosphate'/exp OR 'calcium'/exp) AND ('resin'/exp OR 'tooth cement'/exp OR 'adhesive agent'/exp OR 'dental amalgam'/exp) AND ('demineralization'/exp OR 'remineralization'/exp OR 'dental caries'/exp) | 1105 |
| Google Scholar | (fluoride OR calcium OR phosphate OR glass) AND (composite resin OR amalgam) AND (demineralization OR remineralization OR secondary caries) AND tooth | 100 |
| Clinicaltrials | (fluoride OR calcium OR phosphate OR glass) AND (composite resin OR amalgam) | 89 |
| SIGLE (Open Grey) | (fluoride OR calcium OR phosphate OR glass) AND (composite resin OR amalgam) | 52 |
| Biblioteca de teses e dissertações - CAPES | (fluoride OR calcium OR phosphate OR glass) AND (composite resin OR amalgam) | 551 |

*All searches were conducted on August 21, 2021.

Supplementary Table S2. List of excluded records (and the reason for exclusion) in the full-text eligibility analysis

| # | Study | Publication Title | Reason for exclusion |
| --- | --- | --- | --- |
| 1 | Pani, 2018 | Comparison of high viscosity glass ionomer cement to composite restorations placed in primary teeth under general anesthesia | Study design - observational |
| 2 | Aykut-Yetkiner, 2014 | Comparison of the remineralisation effect of a glass ionomer cement versus a resin composite on dentin of primary teeth | Study design – ex-vivo |
| 3 | Gilmour, 1997 | Prevalence and depth of artificial caries-like lesions adjacent to cavities prepared in roots and restored with a glass ionomer or a dentin-bonded composite material | Study design – in vitro |
| 4 | Donly, 1999 | Evaluating the effects of fluoride-releasing dental materials on adjacent interproximal caries | Study design – in situ |
| 5 | Ziskind, 2007 | Effect of different restorative materials on caries: a retrospective in vivo study | Study design – ex-vivo |
| 6 | Papagiannoulis,  2004 | Clinical evaluation of a polyacid-modified resin composite (compomer) in Class II restorations of primary teeth: a two-year follow-up study | Comparison - absence of control group, with resin or amalgam |
| 7 | Savenau, 2010 | Restorations with composite resin and hybrid materials clinical study for class II cavities | Intervention - used bioactive material together with control, in the same restoration in all groups |
| 8 | Casagrande, 2013 | Randomized clinical trial of adhesive restorations in primary molars. 18-month results | Insufficient data - the study does not show data for separate carious lesions, only general failure |
| 9 | Issa, 2018 | Survival of composite resin and low cost glass ionomer cement for ART: 1 year fo- llow-up of a randomized clinical trial | Insufficient data - the study does not show data for carious lesions, only failure |
| 10 | Diem, 2014 | The effect of a nano-filled resin coating on the 3-year clinical performance of a conventional high-viscosity glass-ionomer cement | Comparison - used bioactive material with resin in the same restoration, in the control group |
| 11 | Gugnani, 2020 | ART with high viscosity GIC and composite restorations in class II cavities: can they thrive in the post-amalgam era? | Study design - the article is a commentary on a previous study (Menezes-Silva, 2019) |
| 12 | Hutchison, 2019 | 10 year comparison of glass ionomer and composite resin restoration materials in class 1 and 2 cavities | Study design - the article is a commentary of other study (Gurgan, 2020) |
| 13 | Kotian, 2020 | Caries of first primary molars requiring glass ionomer cement versus composites as restorative materials | Study design - observational |
| 14 | Naik, 2017 | Comparative evaluation of secondary caries formation around light-cured fluoride-releasing restorative materials | Study design – in vitro |
| 15 | Rożniatowski,  2021 | Clinical study on resin composite and glass ionomer materials in II class restorations in permanent teeth | Insufficient data - reports suspected caries and no presence |
| 16 | Taifour, 2002 | Effectiveness of glass-ionomer (ART) and amalgam restorations in the deciduous dentition: results after 3 years | Insufficient data - it is not clear the number of restores/group |
| 17 | Silva, 2019 | Avaliação de protocolos para remoção seletiva de tecido cariado e para o tratamento restaurador atraumático em molares decíduos: um ano de seguimento de estudos clínicos randomizados | Insufficient data - the study does not show data for separate carious lesions |
| 18 | Rodrigues, 2013 | Avaliação clínica de restaurações classe v com técnica restauradora direta e semidireta | Insufficient data - the study does not report the n for each material |

Supplementary Table S3. Characteristics as reported in the included clinical studies. All reports are described. When a single study was reported in more than one report, they were presented under the same Study ID

| Study ID | Reports* Author, year | Groups  (manufactures) | Tooth  (n) | Follow up (year) | Number of operators | Field Isolation | Study Design as described in report | Criteria | Failed restorations/  total of restorations | Overall risk of bias |
| --- | --- | --- | --- | --- | --- | --- | --- | --- | --- | --- |
| *Randomized Clinical Trials* | | | | | | | | | | |
| ID1 | Adeleke, 2012 | RMGIC (NR)  RC (NR) | permanent  (n=125) | 0.5  1 | 1 | Abs | Randomized | USPHS | 0.5 years  RMGIC= 0/154  RC= 0/86  1 year  RMGIC= 0/117  RC= 0/72 | RoB 2: High |
| ID2 | Akman, 2020 | RC (Filtek Z550, 3M; SonicFill, Ker and X-Tra Fil, Voco)  GIC (Equia, GC) | deciduous  (n=160) | 3 months  0.5  1 | 1 | Rel | Randomized | USPHS | 3 months  RC= 0/110  GIC= 0/37  0.5 years  RC= 0/100  GIC= 0/34  1 year  RC= 0/100  GIC= 0/34 | RoB 2: Some concerns |
| ID3 | Attin, 1998 | RC (TPH-Spectrum, Dentsply)  Compomer (Compoglass, Vivadent) | deciduous  (n=190) | 1 | 3 | Rel | Randomized | Cvar e Ryge | RC= 2/93  Compomer = 4/89 | RoB 2: Some concerns |
|  | Attin, 2000 | RC (TPH-Spectrum, Dentsply)  Compomer (Compoglass, Vivadent) | deciduous  (n=132) | 2 | 3 | Rel | Randomized | Cvar e Ryge | RC= 5/68  Compomer = 3/64 |  |
|  | Attin, 2001 | RC (TPH-Spectrum, Dentsply)  Compomer (Compoglass, Vivadent) | deciduous  (n=190) | 1  2  3 | 1 | Rel | Randomized | Cvar e Ryge | 1 year  RC= 3/96  Compomer= 5/94  2 years  RC= 5/91  Compomer= 3/90  3 years  RC= 2/46  Compemer= 5/46 |  |
| ID4 | Balkaya, 2019 | RC (Charisma Smart, Kulzer and Filtek Bulk Fill, 3M)  GIC (Equia, GC) | permanent  (n=109) | 0.5  1 | 1 | Rel | Randomized | USPHS | 0.5 years  RC = 0/71  GIC= 0/32  1 year  RC = 0/71  GIC= 0/32 | RoB 2: Low |
|  | Balkaya, 2020 | RC (Charisma Smart, Kulzer and Filtek One Bulk Fill, 3M)  GIC (Equia, GC) | permanent  (n=109) | 0.5  1  2 | 1 | Rel | Randomized | USPHS | 1 year  RC = 0/71  GIC= 0/32  2 years  RC = 0/63  GIC= 0/21 |  |
| ID5 | Daou, 2008 | Compomer (Dyract, Dentisply)  RMGIC (Fuji II LC, GC)  GIC (Fuji IX, GC)  AAG (Permite C, SDI) | deciduous  (n=149) | 0.5  1 | 5 | NR | Randomized | USPHS | 05. years  Compomer= 1/39  RMGIC= 0/37  GIC= 1/35  AAG= 0/38  1 year  Compomer= 3/36  RMGIC= 1/33  GIC= 4/33  AAG= 1/36 | RoB 2: Some concerns |
| ID6 | De Moor, 2011 | RC (Herculite XRV, Kerr)  GIC (Ketac Fil, 3M)  RMGIC (PhtocaFil, 3M) | permanent  (n=105) | 0.5  1  1.5  2 | 3 | NR | Randomized | McComb | 0.5 years  RC= 4/30  GIC= 0/30  RM GIC = 3/30  1 year  RC= 5/28  GIC = 0/28  RMGIC= 4/28  1, 5 ano  RC= 6/28  GIC = 2/28  RMGIC= 5/28  2 years  RC= 7/27  GIC = 2/27  RMGIC= 7/27 | RoB 2: Low |
| ID7 | Donly, 1999 | AAG (Tytin, Kerr)  RMGIC (Vitremer, 3M) | deciduous  (n=80) | 0,5  1  2  3 | 1 | Abs | Randomized | USPHS | 1 years  AAG= 3/30  RMGIC= 2/30  2 years  AAG= 4/21  RMGIC= 2/21  3 years  AAG= 6/19  RMGIC= 4/19 | RoB 2: Some concerns |
| ID8 | Dutta, 2001 | RMGIC (Fuji II LC, GC)  AAG (Solila, NOVA) | deciduous  (n=480) | 4 meses  8 meses  1 | 1 | NR | Randomized | USPHS | 4 months  RMGIC= 3/315  AAG= 3/114  8 months  RMGIC= 7/295  AAG= 6/112  1 year  RMCIV= 14/290  AAG=13/100 | RoB 2: High |
| ID9 | El-Housseiny, 2019 | GC (Glass Fill, GCPDental)  RMGIC (Fuji II LC, GC)  RC (Filtek Z250, 3M) | deciduous  (n= 162) | 0,5  1 | 1 | Abs | Randomized | Cvar e Ryge | 1 year  GC = 0/52  RMGIC= 0/53  RC = 0/47 | RoB 2: High |
| ID10 | Ersin, 2006 | RC (Surefil, Dentsply)  GIC (Fuji IX, GC) | deciduous  (n=419) | 0.5  1  2 | 3 | Rel | Randomized | USPHS | 2 years  RC= 29/168  GIC= 37/176 | RoB 2: Low |
| ID11 | Fagundes, 2014 | RC (Tetric, Ivoclar)  RMGIC (Vitremer, 3M) | permanent  (n=70) | 0.5  1  2  5  7 | 1 | Abs | randomized | USPHS | 0.5 years  RC= 0/30  RMGIC= 0/34  1 year  RC= 0/30  RMGIC= 0/35  2 years  RC= 0/26  RMGIC= 0/33  5 years  RC= 2/17  RMGIC= 0/27  7 years  RC= 1/13  RMGIC= 0/23 | RoB 2: Some concerns |
| ID12 | Frencken, 2007 | GIC (Fuji IX, GC and Ketac Molar, 3M)  AAG (Avalloy, Cavex) | permanent  (n=1117) | 6 | 8 | Rel | Randomized | Frencken for ART | GIC= 11/487  AAG= 15/403 | RoB 2: Low |
| ID13 | Gurgan, 2015 | RC (Gradia Direct, GC)  GIC (Equia, GC) | permanent  (n=140) | 1  2  3  4 | 2 | Rel | Randomized | USPHS | 1 year  RC= 0/57  GIC = 0/57  2 years  RC= 0/55  GIC = 0/55  4 years  RC= 0/52  GIC = 0/52 | RoB 2: Low |
|  | Gurgan, 2016 | RC (Gradia, GC)  GIC (Equia, GC) | permanent  (n=140) | 1  2  3  4  5  6 | 2 | Rel | Randomized | USPHS | 1 year  RC= 0/57  GIC= 0/57  2 years  RC= 0/55  GIC= 0/55  3 years  RC= 0/53  GIC= 0/54  4 years  RC= 0/52  GIC= 0/52  5 years  RC= 0/52  GIC= 0/52  6 years  RC= 0/47  GIC= 0/47 |  |
|  | Gurgan, 2020 | GIC (Equia, GC)  RC (Gradia, GC) | permanent  (n=124) | 1  2  3  4  5  6  7  8  10 | 2 | Rel | Randomized | USPHS | 1 year  GIC= 0/68  RC= 0/68  3 years  GIC= 0/64  RC = 0/66  4 years  GIC= 0/62  RC = 0/64  6 years  GIC= 0/57  RC = 0/58  10 years  GIC= 0/61  RC = 0/63 |  |
| ID14 | Jassal, 2018 | RMGIC (Fuji II LC, GC)  RC (Solare-X, GC) | permanent  (n=294) | 0.5  1  1.5 | NR | Rel | Randomized | FDI | 0.5  RMGIC= 0/95  RC= 0/189  1 year  RMGIC= 0/90  RC= 0/181  1,5 years  RMGIC= 0/90  RC= 0/178 | RoB 2: Low |
| ID15 | Kaurich, 1991 | RC (Silux, 3M)  GIC (Ketac Fil, 3M) | permanent  (n=54) | 2 | 4 | Rel | Randomized | NR | 2 years  RC= 1/23  GIC = 1/23 | RoB 2: Low |
| ID16 | Kharma, 2018 | RC (Amelogen Plus, Ultradent)  GIC (Equia, GC) | permanent  (n=40) | 3 months  6 months (0.5 years)  9 months | 1 | Abs | Randomized | USPHS | 3 months  RC= 0/20  GIC= 0/20  6 months (0.5 years)  RC= 0/20  GIC= 0/20  9 months  RC= 0/20  GIC= 0/20 | RoB 2: Some concerns |
| ID17 | Kotsanos, 2004 | RMGIC (Vitremer, 3M)  AAG (Dsipersalloy, Johnson & Johnson)  RC (Spectrum, Dentsply) | deciduous  (n= 82) | 2 | 1 | Abs | Randomized | The modified Pitts [1984] | RMGIC= 5/41  AAG or RC= 5/41 | RoB 2: High |
| ID18 | Koubi, 2006 | RC (IntenS, Vivadent and Filtek Flow, 3M)  RMGIC (Fuji II LC, GC) | permanent  (n=56) | 1 | 3 | NR | Randomized | USPHS | RC = 0/42  RMGIC =0/14 | RoB 2: Some concerns |
| ID19 | Kupietzky, 2019 | RC (Filtek P60, 3M)  GIC (Equia, GC) | deciduous  (n=131) | 3 | 1 | Abs | Randomized | authors' criteria | RC= 2/58  GIC= 0/58 | RoB 2: Some concerns |
| ID20 | Levy, 1990 | RC (Silux, 3M)  GIC (Ketac Fil, 3M) | permanent  (n=104) | 2 | 2 | Rel | Randomized | USPHS | RC= 2/34  GIC= 0/20 | RoB 2: High |
| ID21 | Mandari, 2003 | AAG (ANA 200, Nordiska)  GIC (Fuji II, GC) | permanent  (n=430) | 6 | 1 | Rel | Randomized | USPHS | AAG=16/164  GIC= 3/177 | RoB 2: Low |
| ID22 | McComb, 2002 | GIC (Ketac Fil, 3M)  RMCIV (Vitremer, 3M)  RC (Z100, 3M) | permanent  (n=150) | 0,5  1  1,5  2 | NR | NR | Randomized | McComb | 0.5 year  GIC= 0/38  RMGIC= 1/44  RC= 5/44  1 year  GIC = 0/19  RMGIC= 1/34  RC= 7/36  1.5 years  GIC = 0/7  RMGIC= 1/19  RC= 8/24  2 years  GIC = 0/4  RMGIC= 1/9  RC= 8/18 | RoB 2: Some concerns |
| ID23 | Medeiros, 2015 | RC (Z100, 3M)  RMGIC (Vitremer, 3M) | permanent  (n=60) | 0.5  1 | 1 | Abs | Randomized | USPHS | 0.5 years  RC= 0/30  RMGIC= 0/30  1 years  RC= 0/22  RMGIC= 0/22 | RoB 2: Low |
| ID24 | Menezes-Silva, 2019 | RC (Z350, 3M)  GIC (Equia Fil, GC) | permanent  (n=154) | 0.5  1 | 1 | Rel | Randomized | USPHS  Frencken for ART | 0.5 years  RC= 0/77  GIC= 0/76  1 year  RC= 0/74  GIC= 2/71 | RoB 2: low |
|  | Menezes-Silva, 2021 | GIC (Equia, GC)  RC (Z350, 3M) | permanent  (n=154) | 0.5  1  2 | 1 | Rel GIC  Abs RC | Randomized | USPHS  ART criterion | 0.5 years  GIC= 0/76  RC= 0/77  1year  GIC= 2/71  RC= 0/74  2 year  GIC= 3/62  RC= 2/66 |  |
| ID25 | Miletic, 2020 | RC (Tetric, Ivoclar)  GIC (Equia, GC) | permanent  (n=360) | 1  2 | 8 | Rel GIC  Abs RC | Randomized | FDI | 1 year  RC= 1/162  GIC= 0/162  2 years  RC= 1/143  GIC= 0/143 | RoB 2: Some concerns |
| ID26 | Molina, 2020 | GIC (Equia, GC)  RC (Z250, 3M) | permanent  (n=272) | 0.5  1  2 | 2 | Rel GIC  Abs RC | Randomized | USPHS  ART criterion | 0.5 years  GIC= 0/134  RC= 0/135  1year  GIC= 0/134  RC= 0/135  2 years  GIC= 0/131  RC= 0/134 | RoB 2: Low |
| ID27 | Oliveira, 2017 | RC (Filtek One Bulk Fill, 3M)  RMGIC (Vitremer, 3M) | deciduous  (n=39) | 1 month  6 months | 1 | Abs | Randomized | USPHS | 1 month  RC= 0/18  RMGIC= 1/21  6 months  RC= 0/18  RMGIC= 3/21 | RoB 2: Low |
| ID28 | Ostlund, 1992 | AAG (ANA 2000, ANA)  RC (Occlusion, ICI)  GIC (ChemFil II, Kerr) | deciduous  (n=75) | 3 | 2 | Abs | Randomized | USPHS | AAG= 1/23  RC= 3/19  GIC = 0/10 | RoB 2: High |
| ID29 | OZ, 2020 | GIC (Fuji Bulk, GC)  RC (G-aenial, GC) | permanent  (n=134) | 0.5  1 | 1 | Rel | Randomized | USPHS | 0.5 years  GIC= 0/65  RC= 0/66  1 years  GIC= 0/59  RC= 0/62 | RoB 2: Low |
| ID30 | Ozgunaltay, 2001 | RC (Z100, 3M0  RMGIC (Viteremer, 3M) | permanent  (n=98) | 0.5  1  2  3 | 1 | Rel | Randomized | USPHS | 0.5 year  RC= 0/45  RMGIC= 0/48  1 year  RC= 0/45  RMGIC= 0/48  2 years  RC= 0/40  RMGIC= 0/44  3 years  RC= 0/40  RMCIV= 0/44 | RoB 2: Some concerns |
| ID31 | Pascon, 2006 | Compomer (F200, 3M and Dyract,  Dentsply)  RC (Heliomolar, Vivadent) | deciduous  (n=79) | 0.5  1  1.5  2 | 3 | Abs | Randomized | USPHS | 0.5 years  Compomer= 1/38  RC=4/22  1 year  Compomer= 1/38  RC=7/22  1.5 years  Compomer= 4/38  RC=9/22  2 years  Comp -F200= 11/38  RC=9/22 | RoB 2: Low |
| ID32 | Pollington, 2008 | RC (Pertac II, 3M)  Compomer (Hytac, 3M) | permanent  (n=60) | 0.5  1  3 | 1 | Rel | Randomized | USPHS | 0.5 years  RC= 0/30  Compomer= 0/30  1 year  Resina= 0/30  Compomer= 0/30  3 years  Resina= 0/30  Compomer= 0/30 | RoB 2: High |
| ID33 | Qvist, 1997 | AAG (Dispersalloy, Johnson & Johnson)  GIC (Ketac fil, 3M) | deciduous  (n=1058) | 3 | 14 | Rel | Randomized | NR | AAG= 11/543  GIC= 2/515 | RoB 2: Some concerns |
|  | Qvist, 2004 | GIC (Ketac fill, 3M)  AAG (Dsipersalloy, Johnson & Johnson) | deciduous  (n= 1058) | 8 | 14 | Rel | Randomized | NR | GIC = 7 /515  AAG=15 /543 |  |
| ID34 | Santiago, 2010 | RC (Tetric Ceram, Ivoclar)  RMGIC (Vitremer, 3M) | permanent  (n=70) | 0,5  1  2 | 1 | Abs | Randomized | USPHS | 1 year  RC= 0/30  RMGIC= 0/35  2 years  RC= 0/26  RMGIC= 0/33 | RoB 2: Low |
| ID35 | Santos, 2010 | RMGIC (Vitremer, 3M)  Compomer (Freedom, SDI)  RC (TPH Spectrum, Dentsply) | deciduos  (n=141) | 4 | 2 | Abs | Randomized | USPHS | RMGIC= 5/46  Compomer= 8/51  RC= 6/44 | RoB 2: Some concerns |
| ID36 | Sengul, 2015 | RC (Valux Plus, 3M)  RMGIC (Fufi II LC, GC)  Compomer (Dyract AP, Dentsply)  Gionomer (Beautiful, Shofu) | deciduous  (n= 146) | 2 | 1 | Abs | Randomized | FDI | RC= 1/40  RMGIC= 0/32  Compomer= 4/36  Gionomer=3/38 | RoB 2: Low |
| ID37 | Skartveit, 1994 | AAG (DeTrey Soliloy, Dentsply)  FAAG (Synalloy Fluore, Dentoria) | permanent  (n=830) | 4 | 4 | NR | Randomized | NR | AAG= 86/352  FAAG= 49/352 | RoB 2: Low |
| ID38 | Svanberg, 1992 | AAG (Dispersalloy, Johnson & Johnson)  GIC (Ketac Silver, ESPE) | permanent  (n=36) | 1  2  3 | 1 | Rel AAG  Abs GIC | Randomized | USPHS | 1 year  AAG= 0/18  GIC= 0/18  2 years  AAG= 0/18  GIC= 0/18  3 years  AAG= 3/14  GIC= 0/14 | RoB 2: Some concerns |
| ID39 | Tyas, 1991 | RC (Silux, 3M)  GIC (Fuji II, GC) | permanent  (n=131) | 1  2  3  4  5 | NR | NR | Randomized | NR | 1 year  RC= 2/100  GIC= 0/100  2 years  RC= 5/100  GIC = 0/100  3 years  RC= 11/100  GIC = 2/100  4 years  RC= 11/100  GIC = 2/100  5 years  RC= 11/100  GIC = 2/100 | RoB 2: Some concerns |
| ID40 | Valenzuela, 1994 | AAG (AMALGA C, Busintaal)  FAAG (AMALGA C, Busintaal) +1%SnF | permanent  (n=76) | 2 | 4 | Rel | Randomized | Duperon | AAG= 6/38  FAAG= 1/38 | RoB 2: Some concerns |
| ID41 | van Dijken, 1996 | RC (Pekafill, Bayer)  RMCIV (Fuji II LC, GC)  Compomer (Dyract, Dentisply) | permanent  (n=154) | 3 | NR | NR | Randomized | USPHS | 3 years  RC = 0/52  RMGIC = 0/43  Compomer = 2/51 | RoB 2: Some concerns |
|  | van Dijken, 2001 | RC (Pekafill, Bayer)  Compomer (Dyract, Dentsply)  RMGIC (Fuji II LC, GC) | permanent  (n=154) | 6 | NR | NR | Randomized | USPHS | RC= 1/51  Compomer= 2/49  RMGIC= 0/41 |  |
| ID42 | van Dijken, 2019 | ACTIVA (Activa Bioactive Restorative, Pulpdent)  RC (Ceram X= 82) | permanent  (n=164) | 0.5  1 | 1 | Rel | Randomized | USPHS | 0.5 years  ACTIVA= 0/81  RC= 0/82  1 years  ACTIVA= 3/74  RC= 0/79 | RoB 2: Low |
| ID43 | Vural, 2020 | RC (TPH3 Spectrum, Denstsply)  RMGIC (Riva, SDI) | permanent  (n=110) | 0.5  1  1.5  2  3 | 1 | Rel | Randomized | USPHS | 0.5 years  RC= 0/55  RMGIC= 0/54  1 year  RC=0/52  RMGIC= 0/52  1.5 years  RC= 0/43  RMGIC= 0/48  2 years  RC= 0/43  RMGIC= 0/47  3 years  RC= 0/43  RMGIC= 0/47 | RoB 2: Low |
| ID44 | Welbury, 1991 | AAG (Amalcap, Ivoclar)  GIC (Ketac Fil, 3M) | deciduos  (n=238) | 5 | 2 | Rel | Randomized | USPHS | AAG = 11/119  GIC= 7/119 | RoB 2: Low |
| ID45 | Zanata, 2003 | GIC (Fuji IX, GC)  ZOE (IRM, Dentsply)  RC (Fill Magic, Vigodent) | permanent  (n=757) | 2 | 1 | Rel | Randomized | Frencken for ART | GIC= 4/417  RC= 1/127 | RoB 2: Some concerns |
| *Clinical Studies (non-randomized)* | | | | | | | | | | |
| ID46 | Amorim, 2013 | AAG (Permite, SDI)  GIC (Ketac Molar Easymix, 3M) | deciduous  (n=750) | 2 | 3 | Rel | Non-randomized | ART criterion | AAG= 24/258  GIC= 33/299 | ROBINS-I: Moderate risk |
|  | Hilbert, 2014 | GIC (Ketac Molar Easy Mix, 3M)  AAG (Permite, SDI) | deciduous  (n=750) | 3 | 3 | Rel | Non-randomized | ART criterion | AAG= 3/251  GIC= 7/282 |  |
| ID47 | Burgess, 2004 | RMCIV (Fuji II LC, GC)  Compomer (Dyract AP, Dentsply)  RC (Pertac III, ESPE and Synergy, Coltene) | permanent  (n=120) | 0.5  1  2  3 | 2 | Abs | Non-randomized | USPHS | 3 years  RMGIC= 0/30  Compomer= 0/30  RC= 2/60 | ROBINS-I: Critical risk |
| ID48 | Franco, 2006 | RC (Tetric Ceram, Ivoclar)  RMGIC (Vitremer, 3M) | permanent  (n=70) | 0.5  1  2  5 | 1 | Abs | Non-randomized | USPHS | 1 year  RC= 0/30  RMGIC= 0/35  2 years  RC= 0/26  RMGIC= 0/33  5 years  RC= 2/17  RMGIC= 0/27 | ROBINS-I: Critical risk |
| ID49 | Gallo, 2005 | RC (Silux Plus, 3M)  Compomer (F200, 3M) | permanent  (n=90) | 0.5  1  2  3 | 2 | Abs | Non-randomized | USPHS | 0.5 years  RC= 0/30  Compomer= 0/60  1 year  RC= 0/30  Compomer= 0/60  2 years  RC= 0/30  Compomer= 0/60  3 years  RC= 0/30  Compomer= 0/60 | ROBINS-I: Critical risk |
| ID50 | Haveman, 2003 | GIC (Ketac Fil, 3M)  RMGIC (Vitremer, 3M)  AAG (Tytin, Kerr) | permanent  (n=114) | 0.5  1  2 | 3 | Abs | Non-randomized | Wood 1993 | 2 years  GIC= 8/27  RMGIC= 7/34  AAG= 18/34 | ROBINS-I: Critical risk |
| ID51 | Mjör, 1993 | AAG (Dispersalloy, Johnson & Johnson)  GIC (Ketac Silver, ESPE)  RC (P10, 3M) | permanent  (n=274) | 3  5 | 3 | Abs | Non-randomized | USPHS | 3 years  AAG= 1/32  GIC= 2/32  RC= 5/32  5 years  AAG= 0/26  GIC= 0/26  RC= 4/26 | ROBINS-I: Critical risk |
| ID52 | Namgung, 2013 | RC (NR)  GIC (NR) | permanent  (n=474) | 1 | 3 categories: professors, residents, students | NR | Non-randomized | USPHS | NR | ROBINS-I: Critical risk |
| ID53 | Powell, 1992 | GIC (Ketac Fil, 3M)  RC (Silux Plus, 3M) | permanent  (n=116) | 2 | NR | Rel | Non-randomized | USPHS | GIC= 1/38  RC= 4/34 | ROBINS-I: Serious |
